# Extending the detectable time window of fast protein dynamics using ^1^H_N_ E-CPMG

**DOI:** 10.1007/s10858-025-00470-1

**Published:** 2025-06-30

**Authors:** Dwaipayan Mukhopadhyay, Supriya Pratihar, Stefan Becker, Christian Griesinger

**Affiliations:** 1 Department for NMR-based Structural Biology, Max Planck Institute for Multidisciplinary Sciences, Am Fassberg 11, 37077 Göttingen, Germany; 2 Present Address: Department of Biochemistry and Molecular Biology, Columbia University Medical Center, New York, NY 10032 USA

**Keywords:** NMR, Ubiquitin, Protein dynamics, Relaxation dispersion, Conformational exchange, Peptide flip

## Abstract

Recent advances in high power NMR relaxation dispersion experiments have significantly enhanced our ability to study fast µs timescale motions in proteins, which are crucial for understanding their biological functions. Here, we have extended the detectable time window of such fast dynamics with the development of **e**xtreme power ^1^H **C**arr-**P**urcell-**M**eiboom-**G**ill (^1^H E-CPMG) experiments targeted at the backbone amide protons (^1^H_N_). Using this methodology, artifact-free relaxation dispersion profiles can be obtained up to extreme pulsing conditions with minimal setup effort using commonly used standard NMR hardware. We demonstrate the utility of ¹H E-CPMG on human ubiquitin, revealing that the previously reported peptide flip motion influences a larger region of the protein backbone than previously recognized. Additionally, we directly observed a faster dynamic process at residue T09, aligning with previously predicted pincer mode motion. These findings underscore the effectiveness of ^1^H E-CPMG in extending the temporal resolution at which biologically relevant fast protein dynamics can be studied.

**Supplementary Information:**

The online version contains supplementary material available at 10.1007/s10858-025-00470-1.

## Introduction

Recently, there has been considerable progress in the development of Nuclear Magnetic Resonance (NMR) relaxation dispersion (RD) based techniques to study fast protein dynamics occurring in < 100 µs timescales utilizing high power handling capabilities of modern cryoprobes (Ban et al. 2012, 2013; Pratihar et al. 2016; Smith et al. 2016; Reddy et al. 2018; Schlagnitweit et al. 2018). In most cases, RD techniques targeting elucidation of such fast timescale processes rely upon the application of spin lock fields with a duration of up to 100 ms and magnitudes of ~ 6.4 kHz (^15^N), ~ 16 kHz (^13^C), and ~ 40 kHz (^1^H) to detect dynamics involving ^15^N, ^13^C, and ^1^H nuclei (Smith et al. 2015). These approaches enhance the conventional low power relaxation dispersion experiments by elevating the power levels within the relaxation dispersion encoding elements of the pulse sequences to the respective hard pulse limits for these channels available on the modern cryoprobes. RD experiments often involve the measurement of transverse rotating frame relaxation time (*R*_1ρ_) values of the nuclei of interest with increasing spin lock field strength (Szyperski et al. 1993; Akke and Palmer 1996; Zinn-Justin et al. 1997; Akke et al. 1998). However, due to difficulty in recording reliable measurements utilizing very low spin lock fields, that would cover a range of chemical shifts, an alternative approach to study slower dynamics has been to measure the modulation of transverse relaxation times (*R*_2_) of the nuclei of interest through applying Carr-Purcell-Meiboom-Gill (CPMG) pulse trains with increased pulse repetition rate (Carr and Purcell 1954; Meiboom and Gill 1958; Orekhov et al. 1994; Meekhof et al. 1998). CPMG experiments are usually performed using hard 180° pulse trains, which have traditionally limited its application in characterizing fast protein dynamics requiring higher repetition rates of such high-power hard pulses. Thus, the characterization of fast protein dynamics ranging from ms to µs timescale often requires a careful combination of these two approaches (Mulder et al. 1999). CPMG-based RD measurements are usually performed in a constant-time (CT) fashion (Mulder et al. 2001) utilizing pulse sequence elements, which balance controlled conversion of in-phase (IP) to anti-phase (AP) magnetization (Loria et al. 1999; Tollinger et al. 2001). Design of high power *R*_1ρ_ pulse sequences, which conventionally measure relaxation of only IP magnetization, must take this difference into account, when combining the experimental results with those obtained from CPMG-based RD techniques (Ban et al. 2013).

Recently, an amide ^15^N E-CPMG (extreme CPMG) experiment (Reddy et al. 2018) has been developed as an alternative to the combination of CPMG experiments at low power regime (up to 1–2 kHz) with *R*_1ρ_ experiments at extreme power (up to 6.4 kHz) integrating both regimes into a single measurement. However, as this experiment utilizing extreme powers targets the ^15^N nuclei, it suffers from the inherent challenges with very high heat generation in the sample and associated problems regarding hardware. In addition, for the ^15^N channel, the value of ~ 6.5 kHz as the upper bound for available hard pulse limit, even in the most power-tolerant cryoprobes, restricts the minimum observable timescales to around 25 µs in the best-case scenarios.

For elucidation of dynamics in the protein backbone, an alternative probe has been the ^1^H_N_ nuclei (Ishima et al. 1998), under the assumption that both nuclei comprising the ^1^H_N_−^15^N bond experience the same dynamic processes. In recent years, multiple pulse sequences have been proposed to study slower motions in the protein backbone utilizing low power (routinely up to ~ 2 kHz) ^1^H_N_ CPMG experiments (Ishima and Torchia 2003; Yuwen and Kay 2019). In modern cryoprobes, the ^1^H channels routinely employ radio frequency (RF) fields up to ~ 30–40 kHz for hard pulses. Because of the high gyromagnetic ratio of ^1^H, the generation of heat is much less of a problem in experiments utilizing such extreme powers compared to ^15^N nuclei since less wattage of RF is required for ^1^H (~10 W) than ^15^N (~ 300 W). The use of ^1^H channel effectively enables the detection of processes with a lifetime ranging from ~ 2.5–5.5 µs with the application of extreme RF field strengths. ^1^H_N_ nuclei have been utilized to elucidate fast processes occurring in the protein backbone through high power *R*_1ρ_ experiments (Pratihar et al. 2016; Smith et al. 2016). However, *R*_1ρ_ experiments pose considerable challenges to set up at such extreme power conditions and to ensure reliable calibration of field strength at very low power conditions. Special consideration must be taken to avoid NOE/ROE type transfers between dipolar coupled ^1^H nuclei (Eichmüller and Skrynnikov 2005), requiring measurements at multiple effective tilt angles and longer delay times, resulting in increased experimental time. We propose to apply the E-CPMG approach to the ^1^H_N_ nuclei as a straightforward alternative to the combined low-power CPMG with high-power ^1^H_N_*R*_1ρ_ experiments. The ^1^H_N_ E-CPMG experiment effectively promises a robust measurement of relaxation dispersion curves ranging from ~ 100 Hz to ~ 30–40 kHz in a single experiment, with minimal effort in setting up.

Usually, the method of choice for the detection of dynamics involving backbone ^1^H_N_ nuclei under low frequency CPMG conditions, is described in the literature (Ishima and Torchia 2003). This pulse sequence, utilizing the relaxation compensated (rcINEPT) element (Loria et al. 1999), can be extended to fast pulsing E-CPMG condition with minimal modifications, analogous to the previously described ^15^N E-CPMG experiment. To reduce off-resonance effects and pulse imperfections under such high pulsing limits as well as to avoid potential Hartman-Hahn type transfers, the [0013] phase cycle for the CPMG pulses described in the literature (Yip and Zuiderweg 2004; Long et al. 2008; Jiang et al. 2015; Yuwen and Kay 2019) is employed. However, as mentioned in the literature (Yip and Zuiderweg 2004), this causes the mixing of the transverse and longitudinal ^1^H_N_ magnetizations during CPMG pulses. Subsequently, instead of only being governed by the transverse relaxation rate (*R*_2_), the decay of magnetization during the CPMG period is also governed by the longitudinal relaxation rate (*R*_1_). This phenomenon gives rise to a differential relaxation (*R*_2_– *R*_1_) dependent linear term, henceforth referred to as “linear decay” in this manuscript, leading to an extra negative linear dependence of the measured transverse relaxation rates (*R*_2,eff_) with increasing CPMG frequency (*ν*_CPMG_). When *ν*_CPMG_ reaches values greater than 2 kHz, this linear decay becomes significant (Fig. S1), especially worsening at high magnetic field and low temperatures, as the difference between *R*_2_ and *R*_1_ increases, jeopardizing the accuracy of the measured *R*_2,eff_ values. To correct this artifact, site-specific measurements of ^1^H_N_*R*_1_ values, usually through inversion recovery experiments, are needed (Fig. S1) (Yip and Zuiderweg 2004; Jiang et al. 2015). However, this increases the experiment time drastically at high magnetic fields and low temperatures. Pulse sequence elements have been proposed in the literature (Yuwen and Kay 2019), which correct for this linear decay in the conventional low-power CPMG experiment. We have combined these previous approaches to design an rcINEPT ^1^H_N_ E-CPMG sequence optimized for robust performance under extreme power conditions.

## Materials and methods

### Sample preparation

Perdeuterated and uniformly ^15^N-labeled human ubiquitin was expressed in 100% D_2_O minimal medium with ^15^NH_4_Cl (Eurisotop) as nitrogen source and 1,2,3,4,5,6,6-d_7_-D-glucose (Eurisotop) as carbon source. Protein expression and purification were performed as described previously (Lazar et al. 1997). The sample was back-exchanged with water to ensure 100% back exchange of ^2^H with ^1^H at all labile sites. The protein was dissolved in 20 mM phosphate buffer, pH 6.5, containing 5% D_2_O, 0.05% NaN_3_, and 50 µM DSS. The final protein concentration was 1 mM and the sample was transferred to a 3 mm standard NMR tube for all NMR measurements.

### NMR spectroscopy

NMR experiments were recorded on 600 MHz and 800 MHz Bruker spectrometers equipped with Avance Neo consoles. The 600 MHz spectrometer was equipped with a liquid nitrogen-cooled 5 mm Cryoprobe Prodigy (CPP TCI 600S3 H&F-C/N-D-05s Z ET), whereas the 800 MHz spectrometer was equipped with a standard helium-cooled 5 mm TCI cryoprobe (CP2.1 TCI 800S6 H/C/N/-D-05 XT). Experiments were recorded at 277 K and 292 K (calibrated using a 3 mm thermocouple) with the Variable Temperature (VT) unit set to operate with a standard 670 L/hour gas flow rate, and the Bruker chiller unit (BCU) was set to medium. No higher flow was necessary because only minimal heating was observed.

**Fig. 1:**
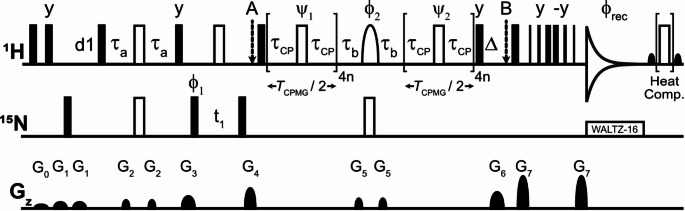
^1^H_N_ E-CPMG pulse sequence for detection of fast chemical exchange processes. The ^1^H carrier is placed at the water resonance, except during the interval between A and B, where it is placed in the middle of the amide ^1^H chemical shift range (at ~ 8 ppm for ubiquitin). Filled bars denote 90° and hollow bars denote 180° RF pulses. The hollow shaped pulse in the rcINEPT element on the ^1^H channel is an amide selective REBURP 180° pulse (4.8 ppm bandwidth for ubiquitin). All pulses have phase *x* unless indicated otherwise. Delays are as follows: d1 = recycle delay, *τ*_a_ = 2.38 ms, *τ*_b_ = 2.68 ms. Compensated CPMG echo delays (*τ*_CP_) and linear decay compensation delay (Δ) are defined in the pulse sequence description and are the same as discussed in the literature (Yuwen and Kay 2019). The phase cycle is ϕ_1_ = x, -x, -x, x, -x, x, x, -x; ϕ_2_ = 4(x), 4(-x), 4(-x), 4(x); ϕ_rec_ = x, -x, -x, x, -x, x, x, -x. Phases ψ_1_ and ψ_2_ are modulated to implement [0013] phase cycle for each successive pulse in the CPMG block (ψ_1_ = y, y, -x, x; ψ_2_ = -y, y, -x, -x), implemented in the same fashion as described in (Yuwen and Kay 2019). In the pulse code itself, during the CPMG encoding in first half, ψ_1_ is incremented, and during the CPMG encoding in second half, ψ_2_ is decremented, starting from the final phase list position reached by ψ_1_. The loopcounter *N* is recommended to be at minimum divisible by 4, thus being 4*n*, where *n* is any positive integer or zero (for the reference plane). For the full phase list and implementation, please refer to the pulse sequence code in Supporting Information. A minimum phase cycle of 4 steps is necessary, although all experiments were recorded with 8 scans. Water suppression is achieved with a 3-9-19 WATERGATE (Sklenar et al. 1993) element. Quadrature detection in the indirect dimension is obtained by incrementing ϕ_1_ according to the STATES-TPPI method (Marion et al. 1989). Optional heat compensation is achieved by applying a variable number of 180° hard pulses on the ^1^H channel after acquisition to maintain a total number of applied 180° hard pulses constant (corresponding to the value for the highest *ν*_CPMG_). Residual water magnetization is brought along the XY plane before heat compensation and returned to the Z-axis afterward by application of water-selective 90° flip-back pulses indicated by bell-shaped pulses on the ^1^H channel. Gradients (G_z_) with strengths (lengths) of G_0_ = 7.5% (1 ms), G_1_ = 15% (1 ms), G_2_ = 20% (0.5 ms), G_3_ = 30% (1 ms), G_4_ = 50% (0.8 ms), G_5_ = 24% (0.5 ms), G_6_ = 40% (1 ms), G_7_ = 80% (0.8 ms) were applied. Gradient strengths are given in percentage of maximum strength

Relaxation dispersion experiments were recorded with the pulse sequence described in Fig. 1. For all experiments, at both spectrometers, high-power pulses were set to operate with 12 W for the ^1^H channel and 150 W for the ^15^N channel. Corresponding to these power levels at the 600 MHz spectrometer, ^1^H 90° high power pulse length and ^15^N 90° high power pulse length were found to be 8.4 µs and 37.5 µs, respectively. At the 800 MHz spectrometer, using the same power levels, the ^1^H 90° high power pulse length and ^15^N 90° high power pulse length were found to be 7.6 µs and 44 µs, respectively. The ^1^H_N_ E-CPMG experiment was encoded as a pseudo-3D dataset, resulting in a stack of ^1^H-^15^N HSQC planes, where the CPMG data points were recorded in an interleaved manner. All CPMG experiments were performed in a constant time (CT) fashion (Mulder et al. 2001), where the first plane in each experiment was a reference plane acquired without any CPMG duration. For all other planes, a constant CPMG time (*T*_CPMG_) of 40 ms was utilized. The CPMG frequencies (*ν*_CPMG_) were increased from 200 Hz to the maximum available value corresponding to the high-power limits of the individual spectrometers and probes. Each experiment at 600 MHz contained 40 CPMG data points (including the reference plane and four repeat points) at 277 K and 24 points (including the reference plane and two repeat points) at 292 K. The dataset recorded at 800 MHz and 277 K contained 28 CPMG points (including the reference plane and three repeat points). Each CPMG data point represented a 2D ^1^H–^15^N plane comprising 128 points along the ^15^N indirect dimension (with a spectral width of ~ 21.92 ppm at 600 MHz and ~ 22.83 ppm at 800 MHz), and 1024 points along the ^1^H direct dimension, going up to ~ 50 ms acquisition time during which low power ^15^N WALTZ-16 (Shaka et al. 1983) decoupling was used. All experiments were recorded with 8 scans and 128 dummy scans. To minimize heating and to preserve hardware integrity, a 3 s recycle delay was used between scans to ensure low duty cycle value. The experimental time was ~ 51 min per CPMG plane. The experiments on the 600 MHz spectrometer were generally performed without heat compensation. However, additional data sets were recorded at the 600 MHz spectrometer. One such dataset was recorded with a heat compensation block to check for any potential effect of sample heating on the measured relaxation rates. Another dataset was recorded where a hard 180° refocusing pulse was used in place of the REBURP pulse in the rcINEPT element on the ^1^H channel. For both these datasets, all other experimental conditions remained the same as those stated earlier. The experiment on the 800 MHz spectrometer exhibited larger imperfections and subsequently the dataset was recorded with heat compensation to rule out the possibility of systematic sample heating-related artefacts contributing to the results, although this was not strictly necessary. An additional low power CPMG data set was collected with the pulse sequence described in Fig. 1 with a maximum CPMG frequency of 2 kHz comprising of 26 total CPMG data points including reference and repeat planes. To test the effect of RF induced coil heating and detuning we performed a pulse calibration experiment using the same ^1^H_N_ E-CPMG pulse scheme and recorded nutation curves at E- CPMG condition.

### Data processing and analysis

All NMR experiments were processed, and peak volumes were extracted with NMRPipe (Delaglio et al. 1995). Transverse relaxation rates (*R*_2,eff_) values were calculated with respect to a reference spectrum collected without the constant CPMG time (*T*_CPMG_) with the following expression, where *I* represents the extracted peak intensity (Mulder et al. 2001)

1 $$\:\begin{array}{c}{R}_{2,eff}\left({\nu\:}_{CPMG}\right)=-ln\left[I\left({\nu\:}_{CPMG}\right)/I\left(ref\right)\right]/{T}_{CPMG}\end{array}$$

Experimental errors for *R*_2,eff_ were calculated from the standard deviations obtained from the repeat data points in the RD profiles. CPMG dispersion curves were fit to a model representing a fast two-state chemical exchange process according to the following expression derived from the simplified Luz-Meiboom model (Luz and Meiboom 1963):

2 $$\:\begin{array}{c}{R}_{2,\text{e}\text{f}\text{f}}={R}_{\text{2,0}}+{\varphi\:}_{\text{e}\text{x}}{\tau\:}_{\text{e}\text{x}}\left[1-4{\nu\:}_{\text{C}\text{P}\text{M}\text{G}}{\tau\:}_{\text{e}\text{x}}tanh\left(\frac{1}{4{\nu\:}_{\text{C}\text{P}\text{M}\text{G}}{\tau\:}_{\text{e}\text{x}}}\right)\right]\end{array}$$

Where, *R*_2,0_ is the intrinsic transverse relaxation rate without chemical exchange contribution, *τ*_ex_ is the timescale of the fast exchange process ($\:{\tau\:}_{\text{e}\text{x}}=\:1/{k}_{\text{e}\text{x}}$, where *k*_ex_ is rate of chemical exchange between the two states), and *ϕ*_ex_ is the population-weighted chemical shift variance given by$\:{\varphi\:}_{\text{e}\text{x}}={p}_{\text{A}}{p}_{\text{B}}{{\Delta\:}\omega\:}^{2}$. Here, Δ*ω* is the difference of angular frequencies pertaining to states A and B with populations denoted by *p*_A_ and *p*_B_, respectively. Chemical shift variance values calculated in angular units can be converted to parts per million squared for removal of field dependence, thus facilitating ease of comparison among datasets collected at varying magnetic field strengths. The required conversion factor, which is the square of the Larmor frequency of the nucleus of interest in angular units ($\:{\omega\:}_{0}^{2}$) has been directly incorporated in the fitting procedure.

The dispersion curves were fit in a residue-specific manner with independent timescales (*τ*_ex_). The dispersion curves collected at 277 K under 600 MHz (24 unique points randomly chosen from 35 available unique points) and 800 MHz (all 24 unique points) were fit jointly in a residue-specific manner. Dispersion curves which showed significant change of *R*_2,eff_ values (> 1 s^− 1^ at 800 MHz field strength) were considered for fitting. All data was fit and plotted using in-house Mathematica scripts. Errors in fit parameters were estimated from errors in *R*_2,eff_ using 500 Monte Carlo runs.

## Results and discussion

The pulse sequence for recording backbone ^1^H_N_-based CPMG experiments under extreme power conditions is shown in Fig. 1. After the INEPT transfer and subsequent ^15^N frequency labeling, the anti-phase 2H_y_N_z_ coherence is generated, which is then subjected to the constant-time CPMG block, divided into two symmetric parts with an rcINEPT element in the middle. As discussed in the literature (Ishima and Torchia 2003), this placement scheme of the CPMG pulses after the ^15^N indirect dimension evolution period (t_1_) reduces ^1^H_N_ cross-relaxation artifacts. The first half of the basic CPMG block implemented here operates on 2H_y_N_z_ coherence. This CPMG block is then followed by a relaxation compensating rcINEPT element (Loria et al. 1999; Ishima and Torchia 2003) employing an amide selective REBURP (Geen and Freeman 1991) 180° refocusing pulse to interchange anti-phase 2H_y_N_z_ coherence into in-phase H_x_ magnetization while also suppressing potential dipole-dipole/chemical shift anisotropy cross-correlated relaxation effects. The necessity and the effect of the REBURP refocusing pulse over using a hard refocusing pulse on the ^1^H channel during the rcINEPT element have been discussed in great detail in the literature (Ishima and Torchia 2003). We have included results from a data set recorded with a hard 180° refocusing pulse instead of the REBURP pulse in the Supporting Information (Fig. S2). As can be seen from the comparison of the respective RD profiles, the use of the hard pulse induces a small pseudo-dispersion effect of magnitude < 1 s^− 1^ spanning the lower values of CPMG frequency. The RD curves at CPMG frequency values higher than 500 Hz are identical. Thus, the use of REBURP pulse on the ^1^H channel in the rcINEPT element is recommended for this pulse sequence. The resulting H_x_ magnetization is subjected to the second half of the constant-time CPMG block. Generation of in-phase H_x_ magnetization during the second CPMG block produces some extra cross-peaks with minimal intensities arising from ^1^H_N_−^1^H_N_ cross-relaxation. The presence of these cross-peaks was found to have negligible effects in the present study. On the other hand, if this becomes a source of significant problems, proposed methods (Ishima and Torchia 2003) can be used to correct the RD curves, or peaks can be removed from consideration for further analysis.

The CPMG pulses are phase cycled according to the modified version of the [0013] phase cycle (Yip and Zuiderweg 2004; Yuwen and Kay 2019) adjusted for the rcINEPT element. Use of this phase cycle usually introduces a linear decay of the measured *R*_2,eff_ with increasing CPMG frequency (Yip and Zuiderweg 2004) through the mixing of longitudinal and transverse relaxation occurring during the CPMG pulses (see Supporting Information for more details). To correct this linear decay experimentally, the weight of the longitudinal relaxation has to be made constant for all CPMG fields. We have used the elegant solution described in the literature (Yuwen and Kay 2019) by adjusting the inter-pulse delay (*τ*_CP_) between CPMG pulses as prescribed to accommodate for longitudinal relaxation during pulsing and incorporating a compensating delay Δ at the end of the CPMG blocks during which the magnetization is longitudinal, i.e., H_z_. The mechanism of this compensation is briefly summarized here. It is evident from (Yip and Zuiderweg 2004) that during the CPMG refocusing pulses with the [0013] phase cycle, the magnetization experiences transverse relaxation during three-quarters of the refocusing pulse length and longitudinal relaxation during one-quarter of the refocusing pulse duration, on average. Thus, for any given CPMG block loop counter in the pulse program read from the list, *N*, the compensated inter-pulse delay (*τ*_CP_) is adjusted as follows: $\:{\tau\:}_{\text{C}\text{P}}=\left({T}_{\text{C}\text{P}\text{M}\text{G}}/4N\right)-({\tau\:}_{90}\times\:0.75)$ and the compensating delay Δ is given by: $\:{\Delta\:}=({N}_{\text{m}\text{a}\text{x}}-N)\times\:{\tau\:}_{90}$. *N*_max_ is the maximum value of *N* from the loop counter list used in the experiment, and $\:{\tau\:}_{90}$ is the length of the 90° hard pulse. Therefore, for the reference plane the compensation delay Δ is maximum. As the number of CPMG blocks increases in the experiment, the compensation delay is redistributed in the echo durations surrounding the CPMG refocusing pulses. This causes the magnetization to experience constant longitudinal relaxation irrespective of the value of *N*, the subsequent effect of which on the amplitude of the magnetization, is already accounted for in the reference spectrum. Thus, the linear decay is removed even with very large values of *N* corresponding to CPMG frequencies leading up to 30 kHz or more. During each loop of the CPMG block under the [0013] phase cycle, the total duration in which the magnetization undergoes pure transverse relaxation is given by *τ*_CPMG_, where $\:{2\tau\:}_{\text{C}\text{P}\text{M}\text{G}}={2\tau\:}_{\text{C}\text{P}}+({\tau\:}_{\text{180}^\circ\:}\times\:0.75)=\left({T}_{\text{C}\text{P}\text{M}\text{G}}/2N\right)$. Here, $\:{\tau\:}_{180^\circ\:}$ denotes the duration of the ^1^H CPMG refocusing pulses being applied at the ^1^H high power limit available to the probe (usually 12 W). The CPMG frequency ($\:{\nu\:}_{{C}{P}{M}{G}}=1/{4\tau\:}_{{C}{P}{M}{G}}=\:N/{T}_{{C}{P}{M}{G}}$) is modulated by decreasing *τ*_CPMG_ via decreasing the compensated echo delay *τ*_CP_, which is achieved by incrementing N in the list. In extreme CPMG conditions, *τ*_CPMG_ approaches the ^1^H 90° hard pulse duration. As the refocusing hard pulses in the CPMG blocks are phase cycled according to the [0013] scheme, minimum number of CPMG blocks on each half can be set to integer multiples of four, especially for low values of CPMG frequencies (< 1 kHz). However, under E-CPMG conditions, where much higher CPMG frequencies are used (up to 30 kHz in this case), the number of CPMG blocks on each half is recommended to be set to integer multiples of 16. This allows the completion of the full super cycle implemented by permutation of the phases in the basic [0013] block (please refer to the phase list in the pulse sequence code in the Supporting Information). Even under such extreme power conditions, the modified CPMG elements proposed earlier (Yuwen and Kay 2019) were found to produce reliable results. This experiment in E-CPMG limit resembles high power R_1ρ_ or the HEROINE (Ban et al. 2013) experiments (both employing extreme power spin lock fields) due to the CPMG duration almost being totally filled with hard pulses. However, because of the modified delays compensating for longitudinal relaxation during [0013] phase cycled pulses, the CPMG block in this ^1^H_N_ E-CPMG pulse sequence never fully converts into a constant phase spin lock field, even under the most extreme CPMG frequency.

The lower requirement of absolute values in wattage to reach the same nutation frequency is one of the biggest advantages of ^1^H-based RD experiments over ^15^N RD experiments, where sample heating poses a significant problem. To further reduce the possibility of sample heating from pulsing at such extreme conditions, maintaining the ionic strength of the sample to low values is recommended by using low salt concentrations in the sample. We have included overlays of 1D ^1^H (Fig. S3A, at 600 MHz) and 2D ^15^N-^1^H (Fig. S3B, at 800 MHz) spectra acquired with the smallest and largest values of CPMG frequency used in the experiments. The spectra overlap within resolution limit and there is no peak shift from sample heating. However, in the case of samples requiring unavoidable higher salt and buffer concentrations, sample heating may still occur as sample heating is generally a function of the electric susceptibility (Wang and Bax 1993). To address this potential issue, we have included an optional heat compensation block, which keeps the total number of 180° hard pulses being applied on the ^1^H channel constant. We have also included a comparison of the effect of this heat compensation block for the 600 MHz spectrometer in the Supporting Information (Fig. S3C). These results indicate that for sample condition described in this manuscript, sample heating is not an issue while reaching extreme power conditions on ^1^H channel using the ^1^H_N_ E-CPMG pulse sequence. To ensure minimal heating, the duty cycle was kept to a very low value using a 3 s recycle delay with a maximum of 40 ms constant CPMG duration (*T*_CPMG_). Monitoring the cryopanel heater reserve power indicated that overall heat generation in the probe head was more sensitive to low-power ^15^N decoupling being used during the acquisition than the E-CPMG ^1^H pulses being applied, the contribution of which was negligible. To reduce the impact of required low power ^15^N decoupling on probe heating, the acquisition time for direct ^1^H detection dimension was set to a maximum of 50 ms. However, for cases where higher ^1^H resolution is necessary, a potential trade-off of larger acquisition time can be implemented with increased recycle delay with reduced *T*_CPMG_. *T*_CPMG_ can also be reduced by increasing the contribution of fast chemical exchange (*R*_ex_) to the measured overall transverse relaxation rate (*R*_2,eff_). *R*_ex_ increases quadratically with the magnetic field (*B*_0_) and is inversely proportional to the kinetic rate of fast chemical exchange, which in turn decreases with reduction of sample temperature. Thus, moving to a lower temperature or a higher *B*_0_ field could assist in increasing the exchange contribution to transverse relaxation and subsequent lowering of acceptable *T*_CPMG_ value. In addition, nutation curves were collected at CPMG frequencies of 2 kHz and 30 kHz to check for detuning related pulse length change upon use of extreme power for 40 ms. The results showed a decrease in pulse length of ~ 4% (7.5 µs 90° for 2 kHz vs. 7.2 µs 90° for 30 kHz) for the 800 MHz cryoprobe and showed an increase of ~ 2.6% (8.15 µs 90° for 2 kHz vs. 8.38 µs 90° for 30 kHz) for the 600 MHz Prodigy cryoprobe. Nutation experiments were recorded at two more intermittent power levels, the results of which indicated a linear change. Thus, we can conclude that the effect of detuning is small even for using such extreme power levels and is expected to be smaller than the radio frequency field inhomogeneity that is usually in the low two digit % range. Moreover, this experiment as well as many other experiments developed in the last decade utilizing E-CPMG and high power spinlock fields (summarized in the Introduction), starting from (Ban et al. 2012), clearly demonstrate that, after consultation with hardware vendors, commercially available modern cryoprobes can be safely operated beyond the usual specifications without any short and long-term damage or loss of sensitivity to the probe. This study highlights the benefits of further investigation and reevaluation of the specifications of commonly used commercially available cryoprobes.

The pulse sequence was tested on a uniformly ^15^N labeled ubiquitin sample, which was perdeuterated at all non-exchangeable ^1^H sites. Ubiquitin was chosen because ns to ms motion of the protein backbone had been studied with various NMR experiments (Tjandra et al. 1995; Mills and Szyperski 2002; Briggman and Tolman 2003; Massi et al. 2005; Lakomek et al. 2008; Lange et al. 2008; Hansen et al. 2009; Ban et al. 2011; Charlier et al. 2013; Smith et al. 2016; Wardenfelt et al. 2021), as well as molecular dynamics simulations (Lindorff-Larsen et al. 2016; Champion et al. 2024). Recently, using high power ^1^H_N_*R*_1ρ_ sequence, a concerted peptide flip motion on timescales of tens of µs was detected for ubiquitin at 277 K (Smith et al. 2016). In addition, the terminal residues of ubiquitin exhibit only fast motion below the µs-ms timescale at such ambient temperature conditions. They could, therefore, effectively serve as internal controls for evaluating the performance of the pulse sequence. The necessity of perdeuteration in ^1^H_N_ CPMG experiments has been discussed in the literature (Ishima and Torchia 2003). It eliminates ^3^J(H_N_–H_α_) coupling, while also drastically suppressing the cross-peaks and artifacts arising from ^1^H−^1^H cross-relaxation. Perdeuteration leads to a reduction of intrinsic relaxation rates (*R*_2,0_) of the ^1^H_N_ nuclei, thus facilitating the detection of smaller magnitudes of underlying chemical exchange processes. Especially, under high power E-CPMG conditions, perdeuteration coupled with the [0013] phase cycle suppresses the possibility of H_N_−H_α_ Hartmann−Hahn transfer, even beyond 30 kHz of CPMG frequency, as observed. We would like to point out that this approach, in its current form, may not work with fully protonated proteins and for highest power E-CPMG condition, presently, perdeuteration remains necessary.

We first tested the performance of this sequence in ubiquitin under ordinary CPMG experimental conditions by acquiring site-specific backbone ^1^H_N_ CPMG dispersion curves up to *ν*_CPMG_ values of 2 kHz at 600 MHz, 277 K (Fig. S4). At this experimental condition, the majority of residues produce flat dispersion profiles free from significant artifacts. For certain residues (F04, V05, K06, G10, E34, D58, Y59, and N60), small oscillations and pseudo-dispersion build-up artifacts (< 1 s^− 1^) were observed for low values of *ν*_CPMG_ < 500–1000 Hz, potentially arising from ^1^H_N_– ^1^H_N_ cross-relaxation as previously discussed in the literature for ^1^H_N_ CPMG and R_1ρ_ experiments (Ishima and Torchia 2003; Eichmüller and Skrynnikov 2005). Relaxation dispersion profiles with significant amplitudes (> 2 s^− 1^) were observed for residues I23, K33, I36, E51, T55, and I61. Most of these residues are known to undergo fast chemical exchange processes as shown by earlier literature (Massi et al. 2005; Hansen et al. 2009; Smith et al. 2016). However, because of the observed low amplitude of the RD curve over this very small *ν*_CPMG_ range compared to the timescale of the underlying processes, we did not fit the dispersion profiles quantitatively. Overall, this result underlines the need to push the *ν*_CPMG_ values to the highest available limit.

**Fig. 2:**
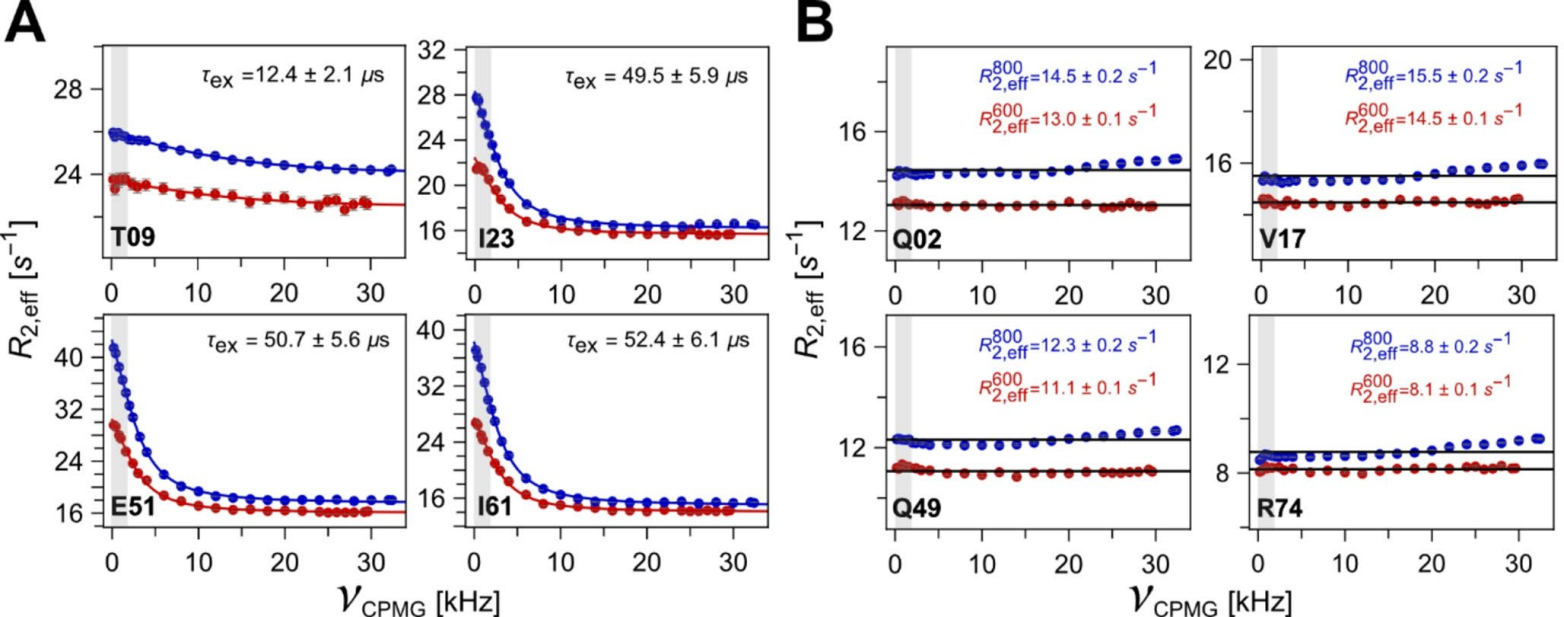
Representative site-specific backbone ^1^H_N_ E-CPMG relaxation dispersion (RD) profiles of ubiquitin obtained using the pulse sequence shown in Fig. 1 at 277 K, 600 MHz (red) and 800 MHz (blue) spectrometers. Grey-shaded areas indicate the regions typically observable with conventional low power CPMG experiments. (**A**) RD profiles for selected residues exhibiting fast µs dynamics with the fit values of site-specific motional timescales indicated inside the figures. (**B**) RD profiles from sites lacking fast detectable dynamics. Black lines indicate the average *R*_2,eff_ values computed over the whole CPMG frequency range. The same average *R*_2,eff_ values, along with computed RMSDs are represented in the figure, color-coded according to magnetic field strength

We then proceeded to record the RD profiles of ubiquitin with the ^1^H_N_ E-CPMG pulse sequence under extreme CPMG conditions at 600 MHz and 800 MHz spectrometers at 277 K. We chose to limit this study to 600 MHz and 800 MHz magnetic field strengths because they are in widespread use. The dispersion profiles collected using the proposed pulse sequence for selected ^1^H_N_ sites are shown in Fig. 2. The dispersion curves for all the resolved residues are shown in Fig. S5. Representative backbone ^1^H_N_ E-CPMG relaxation dispersion profiles from residues exhibiting the presence of unambiguous fast chemical exchange processes recorded with the proposed ^1^H_N_ E-CPMG pulse sequence are shown in Fig. 2A. Dispersion profiles of all residues showing such chemical exchange are shown in Fig. S5A. Many of these residues were previously reported to be involved in peptide-flip motion of the ubiquitin backbone occurring at ~ 55 µs at 277 K, detected using high power ^1^H_N_*R*_1ρ_ experiment (Smith et al. 2016). The datasets acquired at both magnetic fields were simultaneously fit to the motional model under fast-exchange approximation, producing a single *τ*_ex_ and independent *ϕ*_ex_ values for each B_0_ field in a residue-specific fashion. We note here that *τ*_ex_ was fit in a per residue fashion and fitting for a single global *τ*_ex_ corresponding to the underlying motional mode was not performed to keep the focus of this paper on the pulse sequence performance. The fitted *τ*_ex_, *ϕ*_ex_, values are included in the figure panels in Fig. S5A. Compared to the high power ^1^H_N_*R*_1ρ_ experiment (Smith et al. 2016), dispersion profiles with larger amplitudes and less scatter were obtained at 600 MHz. The RD curves of D58 and Y59 under 600 MHz showed a small rising pseudo-dispersion artifact (< 1 s^− 1^) within *ν*_CPMG_ values of 500 Hz. Utilization of the 800 MHz spectrometer with the E-CPMG pulse sequence enabled the collection of RD profiles with larger variations in *R*_2,eff_ values, producing evidence of fast timescale peptide-flip motion impacting more residues (T22, A28, K29, I30, G35, Q40, Q41, A46, G47, K48, L56, D58 Y59, Q62, V70, and R72), than reported earlier. For some residues (Q40, K48, Y59, and V70), 600 MHz E-CPMG dispersion data was not sufficient to produce meaningful fit results as modulations of *R*_2,eff_ values were less than 1 s^− 1^. However, if the modulation of *R*_2,eff_ values were larger than 1 s^− 1^ for the 800 MHz dataset, the results at both fields were included for fitting.

For all other residues, previously not found to be involved in peptide-flip motion (Smith et al. 2016), the new ^1^H_N_ E-CPMG experiment produced flat dispersion profiles (Fig. 2B for representative residues and Fig. S5B for all resolvable residues), indicating a full in-experiment correction of linear decay even at very high E-CPMG frequencies up to 30 kHz. For these residues, we have indicated the average *R*_2,eff_ values in the figures with the calculated RMSD of *R*_2,eff_ over the whole E-CPMG frequency range showing minimal linear decay or other artifacts. Residues F04, V05, and N60 show some minute (< 1 s^− 1^) cross-relaxation based oscillations and pseudo-dispersion build-up artifacts within frequency ranges of 500 Hz (Fig. S5B). The same result has already been seen for the 2 kHz experiment. However, even for these residues the dispersion profiles are constant for *ν*_CPMG_ values above 1 kHz, indicating that there is no fast exchange detectable at 277 K and that the removal of the linear decay works. A number of residues (T07, L08, I13, D32, E34, D52, E64, L67, L69, and L71) show the presence of relaxation dispersion. However, because of the limited amplitude of the exchange term on *R*_2, eff_ values (< 1 s^− 1^ at 800 MHz), such residues were not considered for motional analysis under present conditions. We observed a very small systematic rise of measured *R*_2,eff_ values for the high values of *ν*_CPMG_ > 22 kHz for the data collected at 800 MHz spectrometer. We have no explanation for this phenomenon. A rise in the sample temperature can be safely excluded since this experiment was recorded with an active heat compensation block, which kept the number of CPMG pulses constant for the whole experiment, irrespective of the CPMG frequency. However, as noted, this effect is very small.

We note here that in addition to the peptide-flip motion directly observed previously as well as in this report, a faster motion has been predicted to impact a smaller number of residues around β_1_−β_2_ loop and more sites distributed throughout the ubiquitin backbone, termed as “pincer” mode (Lange et al. 2008). Existence of this motional mode has been probed with additional experiments (Charlier et al. 2013; Michielssens et al. 2014; Wardenfelt et al. 2021; Champion et al. 2024). The specific characterization of timescale and magnitude pertaining to this motion has been subject to debate because of apparent disagreement among the results obtained from various methods and the lack of directly observed unambiguous relaxation dispersion data. Using this new high power ^1^H_N_ E-CPMG experiment, for residue T09 (Fig. 2A), we unambiguously observe the presence of a faster motion compared to peptide-flip mode, which is found to fit to a timescale of ~ 12.5 µs at 277 K. We will describe the nature of this motion in further work.

**Fig. 3:**
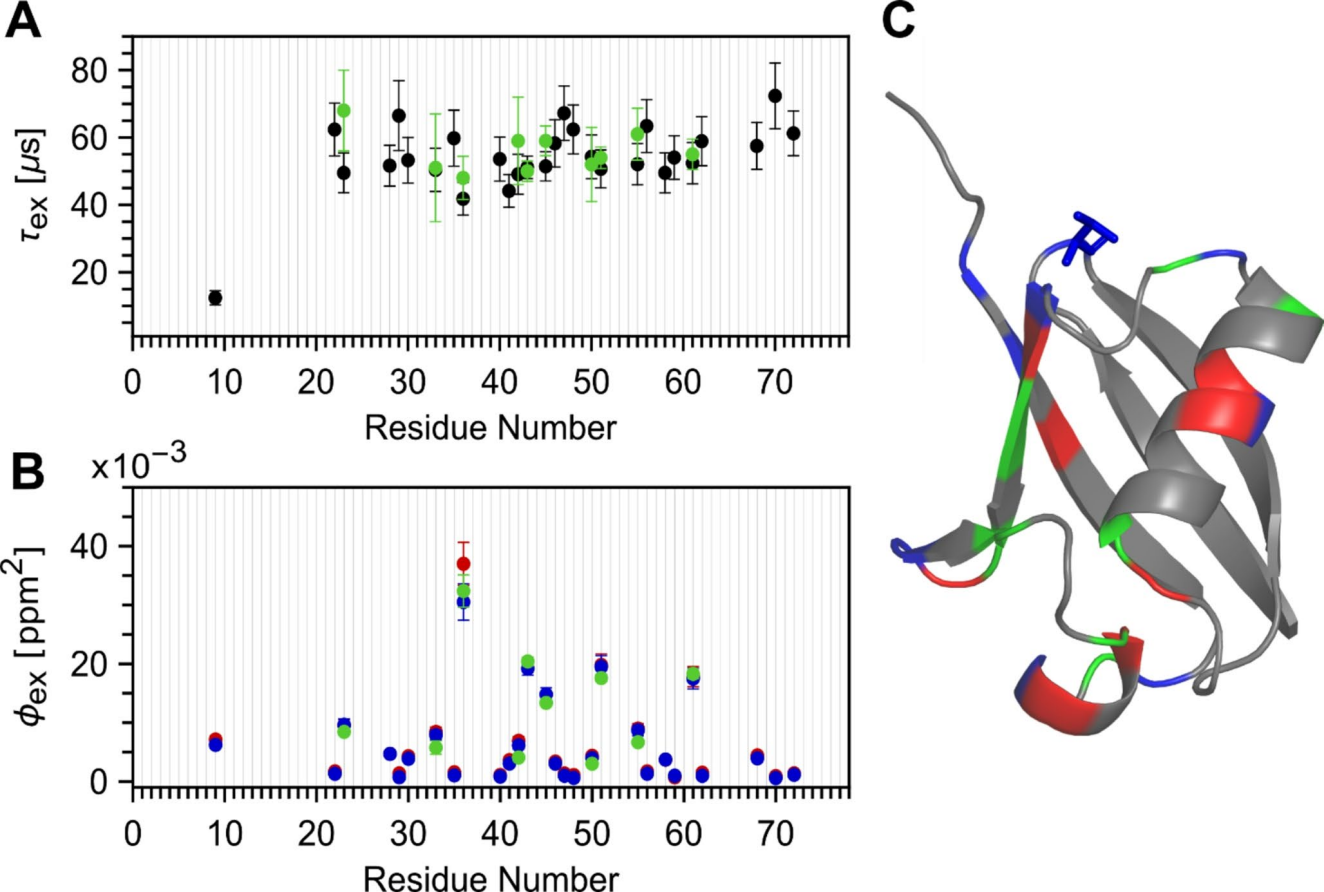
Comparison of residue-specific fit parameters derived from ^1^H_N_ E-CPMG pulse sequence shown in Fig. 1 with previously published results (Smith et al. 2016) obtained using high power ^1^H_N_*R*_1ρ_ pulse sequence. (**A**) Comparison of the fitted timescales (*τ*_ex_) from current experiments (black) with previously published results (green). (**B**) Comparison of fitted chemical shift variances (*ϕ*_ex_) from current experiments at 600 MHz (red) and 800 MHz (blue) with previously published results (green). (**C**) Cartoon representation of ubiquitin (PDB ID: 1UBQ) showing the extent of fast motion present through the backbone reported in the earlier study (green) with newly detected extra sites using current experiment at 600 MHz (red) and 800 MHz (blue). T09, which is found to be participating in a faster motion, is represented as blue sticks

These fitted timescales (*τ*_ex_) pertaining to peptide flip and a faster mode (in case of residue T09) motions (shown in black in Fig. 3A) and corresponding chemical shift variances (*ϕ*_ex_) (shown in Fig. 3B), obtained under both 600 (red in Fig. 3B) and 800 MHz (blue in Fig. 3B) magnetic fields, were compared in Fig. 3 to the previously reported values observed from high power *R*_1ρ_ experiments (Smith et al. 2016) obtained at 600 MHz (green in Fig. 3A and B). Overall, the results obtained using the new experiment show excellent agreement with the previously published results reproducing peptide-flip motions for residues clustered around 50 to 55 µs. Additionally, the new ^1^H_N_ E-CPMG pulse sequence coupled with the application of higher field enabled the discovery of more residues involved in peptide-flip motion, as summarized in Fig. 3C.

**Fig. 4:**
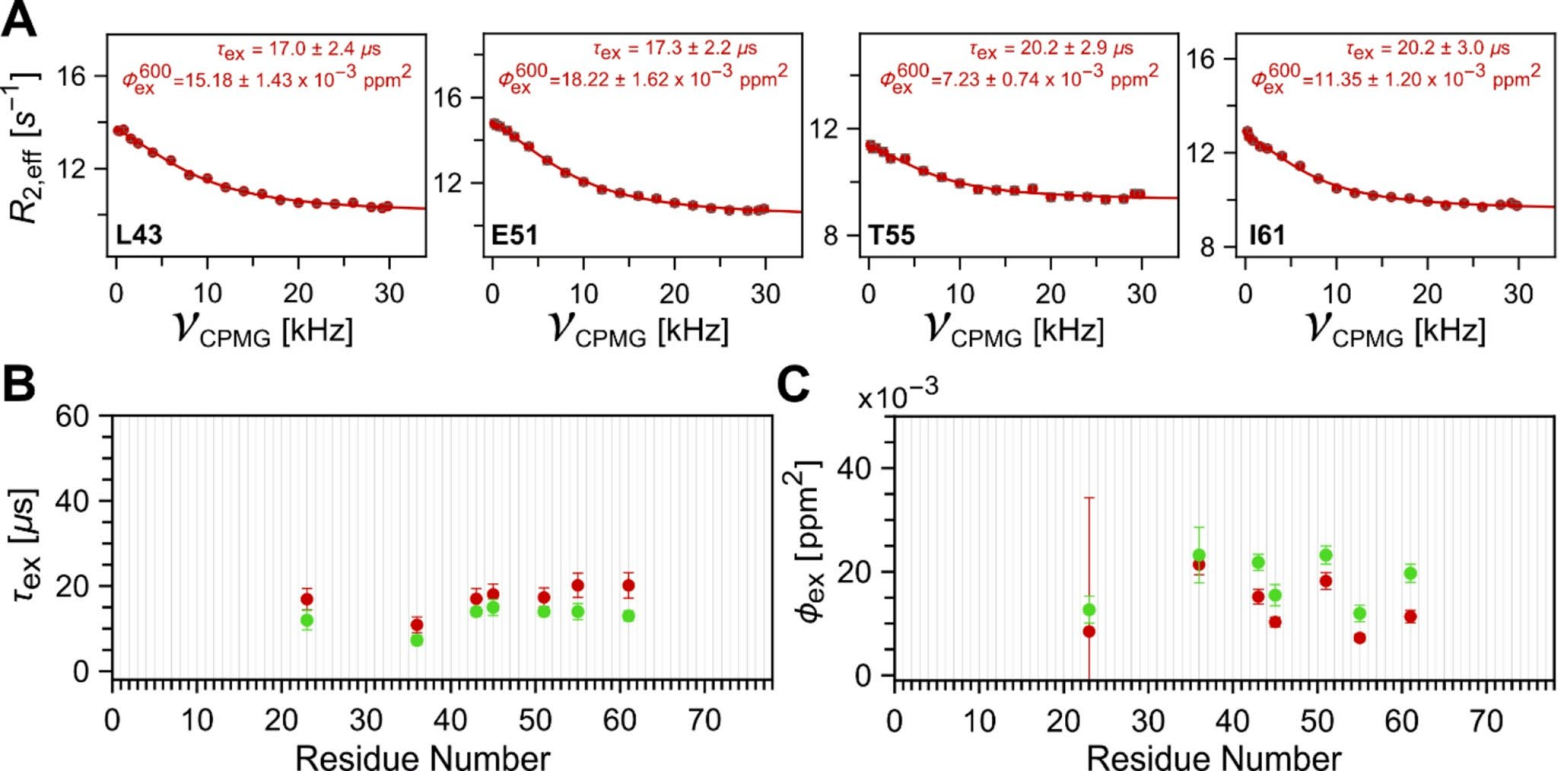
Relaxation dispersion (RD) profiles and results obtained with application of ^1^H_N_ E-CPMG pulse sequence shown in Fig. 1 for ubiquitin backbone ^1^H_N_ sites involved in observable peptide flip motion measured at 292 K and 600 MHz. (**A**) Representative site-specific backbone ^1^H_N_ E-CPMG RD profiles of ubiquitin obtained using the pulse sequence shown in Fig. 1 (red). (**B**) Comparison of fitted timescales (*τ*_ex_) and (**C**) chemical shift variances (*ϕ*_ex_) from data obtained using the pulse sequence shown in Fig. 1 (red) with previously published results (Smith et al. 2016) obtained using high power ^1^H_N_*R*_1ρ_ pulse sequence (green)

Further, to test the limits of this ^1^H_N_ E-CPMG experiment, datasets were recorded at 292 K and 600 MHz, mirroring the experimental condition reported earlier (Smith et al. 2016). The resulting dispersion curves for some representative residues undergoing fast exchange are shown in Fig. 4A, whereas the RD curves for all resolved residues are shown in Fig. S6. Most residues (Fig. S6A), at this temperature, show no dispersion. No significant artifacts are observed while having flat curves with low RMSD, indicating the robustness of this pulse sequence at very high RF power. Clean dispersion profiles are observed for residues I23, I36, L43, F45, E51, T55, and I61 (Fig. S6B) involved in peptide-flip mode, albeit with lower amplitude. Individual fits of the RD profiles were performed for these residues, the fitting details being indicated in the figure legends. Figure 4B and C summarize the fitted timescales (*τ*_ex_) and chemical shift variances (*ϕ*_ex_) along with the previously published results from high power ^1^H_N_*R*_1ρ_ experiments. Overall, there is excellent agreement between the current observed result and previously reported values, with a broad agreement of the extracted timescales clustered around ~ 15 µs with the previously reported global timescale of ~ 13 µs. We note that at higher temperatures, solvent exchange rates at ^1^H_N_ sites increase. Ubiquitin is known to have very large water exchange rates for particular ^1^H_N_ sites, e.g., T09, A46, G75, etc., at elevated temperatures (Rennella et al. 2014). For these residues, the *R*_2,eff_ reported by this experiment includes the contribution of water exchange, producing larger constant *R*_2,eff_ values which are unmodulated by the changing values of CPMG frequency. An increase in estimated noise from repeat point measurements for these residues has also been observed at elevated temperatures (Fig. S6).

## Conclusion

We have introduced a robust ^1^H_N_ E-CPMG experiment capable of detecting protein backbone dynamics on time scales as fast as ~ 5 µs, provided the exchange contribution to relaxation is sufficiently large. Most importantly, by incorporating the previously prescribed correction technique (Yuwen and Kay 2019), this approach eliminates CPMG frequency-dependent linear decay in RD profiles arising from the differential weighting of longitudinal and transverse relaxation, even under extreme pulsing conditions. The ^1^H_N_ E-CPMG experiment serves as a reliable and time-efficient alternative to high-power *R*_1ρ_ measurements for ¹H_N_ nuclei, requiring only minimal setup, primarily careful calibration of ^1^H and ^15^N 90° pulses. This proposed methodology is free from artifacts, producing reliable results within a very reasonable experimental time at commonly available magnetic fields with widely used commercially available cryoprobes and spectrometer hardware without the need for any special setup. Applying this method, we have successfully reproduced previously reported fast exchange parameters for backbone ^1^H_N_ sites in ubiquitin, which are involved in peptide-flip motion. Compared to the previously recorded ^1^H_N_ high-power *R*_1ρ_ measurements, with ^1^H_N_ E-CPMG we observed cleaner RD profiles with reduced scatter and larger amplitudes, while utilizing less experimental time, allowing us to identify additional protein backbone sites engaged in this motion. Furthermore, the application of this pulse sequence has unambiguously revealed the presence and the kinetics of an even faster motion within the β_1_−β_2_ loop region, previously predicted to participate in pincer-mode motion, particularly in residue T09, enabling us to characterize its timescale and chemical shift variance, providing deeper insights into the dynamic landscape of ubiquitin. The enhanced understanding of fast µs protein dynamics by applying ^1^H_N_ E-CPMG experiments on other proteins will significantly improve our grasp of protein function, offering profound insights into the intricate relationship between protein dynamics and their biological roles. Additionally, this methodology can be very easily adapted to study dynamics in the imino protons of DNA and RNA, significantly expanding the scope of its application.

## Electronic supplementary material

Below is the link to the electronic supplementary material.

**Supplementary Material 1**: Supporting information contains details for ^1^H_N_ E-CPMG sequence with uncorrected linear decay from *R*_1_ contribution along with measurement of backbone ^1^H_N_ site-specific relaxation dispersion curves of ubiquitin up to 30 kHz CPMG frequency obtained using the linear decay uncorrected version of pulse sequence (Fig. **S1**); comparison of site-specific backbone ^1^H_N_ E-CPMG relaxation dispersion curves of ubiquitin acquired using hard pulse vs. amide selective REBURP refocusing pulse in the rcINEPT element at 277 K, 600 MHz (Fig. **S2**); overlay of 1D and 2D spectra collected at 600 MHz and 800 MHz with smallest and largest values of CPMG frequency, and comparison of site-specific ^1^H_N_ E-CPMG relaxation dispersion curves of ubiquitin acquired with and without activated heat compensation element at 277 K, 600 MHz (Fig. **S3**); measurement of backbone ^1^H_N_ site-specific relaxation dispersion curves of ubiquitin up to 2 kHz CPMG frequency obtained using the pulse sequence shown in Fig. 1 (Fig. **S4**); measurement of site-specific backbone ^1^H_N_ E-CPMG relaxation dispersion curves of ubiquitin measured up to 30 kHz CPMG frequency at 277 K, 600 MHz and 800 MHz from all resolved sites (Fig. **S5**); measurement of site-specific backbone ^1^H_N_ E-CPMG relaxation dispersion curves of ubiquitin measured up to 30 kHz CPMG frequency at 292 K, 600 MHz from all resolved sites (Fig. **S6**).

## Data Availability

All data are deposited in 10.17617/3.LRPKRE.
