# Supplementary material for: Extending the detectable time window of fast protein dynamics using 1HN E-CPMG
Source: J Biomol NMR. 2025 Jun 30;79(4):231–41. doi: 10.1007/s10858-025-00470-1 (PMC12664843; doi:10.1007/s10858-025-00470-1)
Supplement: Supplementary file 1 — Supplementary Material 1: Supporting information contains details for 1HN E-CPMG sequence with uncorrected linear decay from R1 contribution along with measurement of backbone 1HN site-specific relaxation dispersion curves of ubiquitin up to 30 kHz CPMG frequency obtained using the linear decay uncorrected version of pulse sequence (Fig. S1); comparison of site-specific backbone 1HN E-CPMG relaxation dispersion curves of ubiquitin acquired using hard pulse vs. amide selective REBURP refocusing pulse in the rcINEPT element at 277 K, 600 MHz (Fig. S2); overlay of 1D and 2D spectra collected at 600 MHz and 800 MHz with smallest and largest values of CPMG frequency, and comparison of site-specific 1HN E-CPMG relaxation dispersion curves of ubiquitin acquired with and without activated heat compensation element at 277 K, 600 MHz (Fig. S3); measurement of backbone 1HN site-specific relaxation dispersion curves of ubiquitin up to 2 kHz CPMG frequency obtained using the pulse sequence shown in Fig. 1 (Fig. S4); measurement of site-specific backbone 1HN E-CPMG relaxation dispersion curves of ubiquitin measured up to 30 kHz CPMG frequency at 277 K, 600 MHz and 800 MHz from all resolved sites (Fig. S5); measurement of site-specific backbone 1HN E-CPMG relaxation dispersion curves of ubiquitin measured up to 30 kHz CPMG frequency at 292 K, 600 MHz from all resolved sites (Fig. S6). [file 10858_2025_470_MOESM1_ESM.pdf]

Extending the detectable time window of fast protein dynamics using $^{1}H_{N}$ E-CPMG

Dwaipayan Mukhopadhyay, Supriya Pratihar[†], Stefan Becker, and Christian Griesinger*

Department for NMR-based Structural Biology, Max Planck Institute for Multidisciplinary Sciences, Am Fassberg 11, 37077, Göttingen, Germany

Corresponding author: Prof. Christian Griesinger, Department for NMR-based Structural Biology, Max Planck Institute for Multidisciplinary Sciences, Am Fassberg 11, 37077, Göttingen, Germany, cigr@mpinat.mpg.de

[†]Current address: Department of Biochemistry and Molecular Biology, Columbia University Medical Center, New York, NY 10032, United States

Details related to $^{1}H_{N}$ E-CPMG relaxation dispersion experiment without in-experiment correction of linear decay from R_1 contribution

The $^{1}H_{N}$ E-CPMG relaxation dispersion (RD) experiment without in-experiment correction for longitudinal relaxation contribution related linear term arising from the [0013] phase cycle (abbreviated as linear decay in this manuscript) was performed at 600 MHz, 277 K for comparison. The details and instrumental implementation of the pulse sequence used for this experiment has been discussed elsewhere (Pratihar 2022). In short, this pulse sequence is a straightforward adaptation of the sequence found in (Ishima and Torchia 2003) which has been modified with a [0013] phase cycled CPMG block (Yip and Zuiderweg 2004) but without the compensating delays implemented in (Yuwen and Kay 2019). This dataset was recorded with otherwise identical NMR experimental parameters as discussed in the main text. The RD profiles thus recorded were severely impacted by linear decay under E-CPMG conditions and any meaningful data analysis from such RD trajectories required thorough analytical corrections. Previous literature provides an analytical expression (Yip and Zuiderweg 2004) for such correction. However, this necessitates recording of additional datasets through the following experiment. Site-specific $^{1}H_{N}$ R_1 values were obtained by recording an additional pseudo-3D dataset, which included an interleaved inversion recovery element with variable inversion recovery delays preceding a standard ^{1}H - ^{15}N HSQC readout block. This experiment was recorded with 20 points in the $^{1}H_{N}$ dimension using a long (10 s) recycle delay. To analytically correct the RD profiles with the obtained site-specific R_1 values, the following equation derived from the literature (Yip and Zuiderweg 2004) was used:

$$R_{2,\mathrm{eff}} = R_2 + \frac{(R_2 - R_1)\tau_{180}}{4\tau_{\mathrm{CPMG}}}$$

Here, $R_{2,\text{eff}}$ is the measured uncorrected transverse relaxation rate from CPMG experiment containing contribution of modulation by linear decay. R_2 and R_1 are the true transverse and longitudinal relaxation rates. The other symbols have the same meaning as described in the main manuscript.

The resulting analytically corrected RD profiles along with the uncorrected ones are also shown in Figure S1.

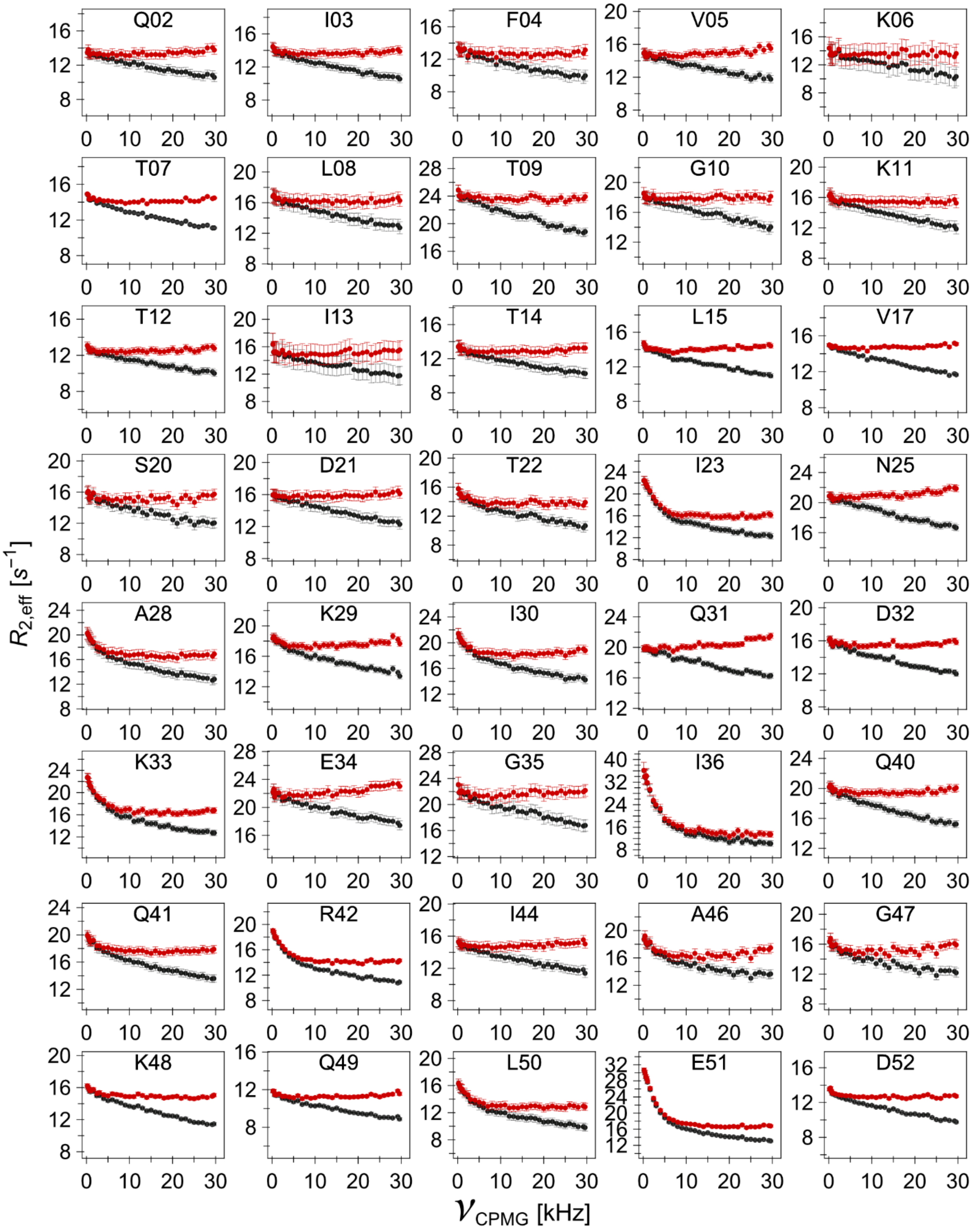

Figure contd.

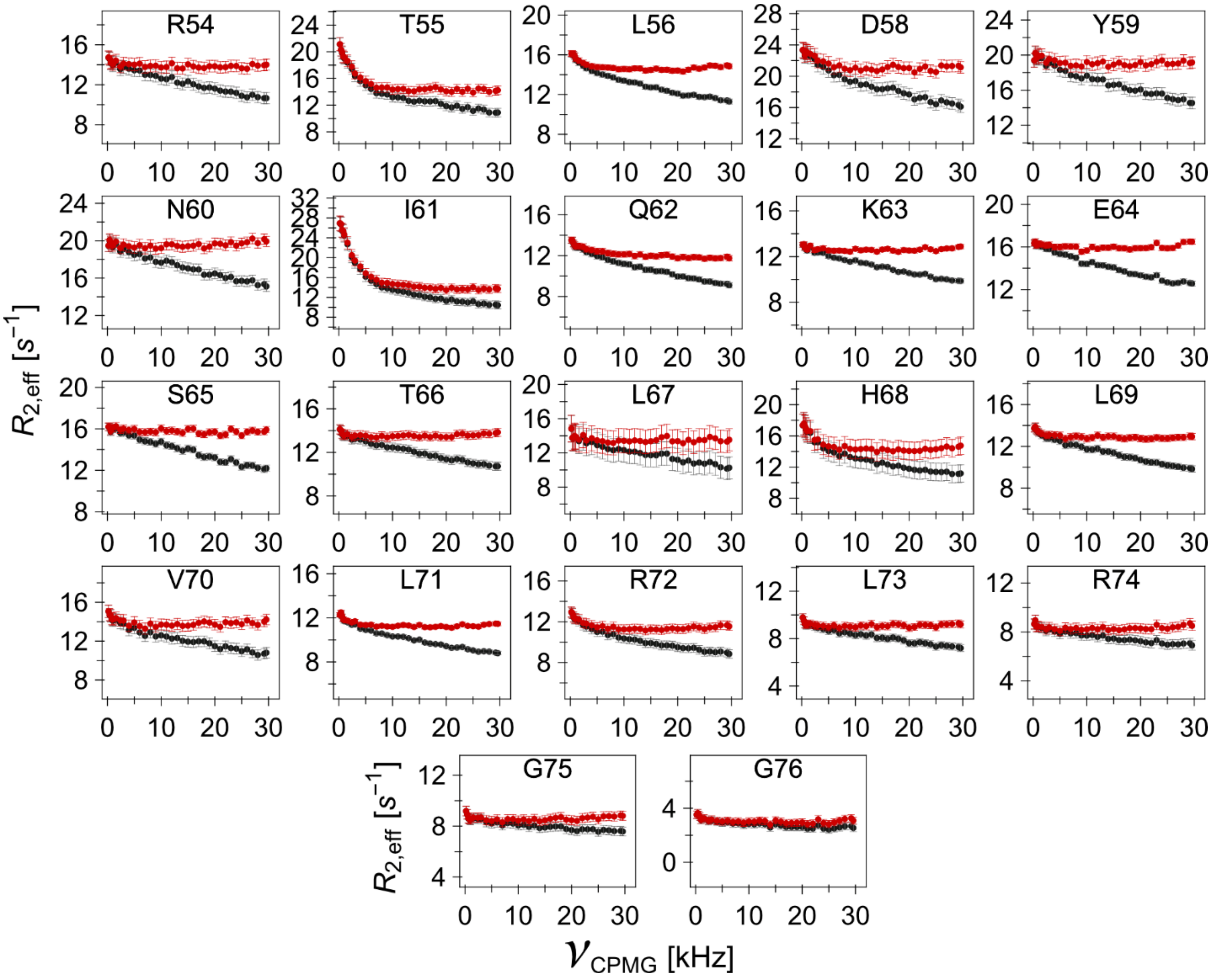

Fig. S1 Measurement of backbone $^{1}H_{N}$ site-specific relaxation dispersion curves of ubiquitin up to 30 kHz CPMG frequency obtained using the linear decay uncorrected version of pulse sequence (black) and analytically corrected dispersion profiles (red) at 600 MHz, 277 K.

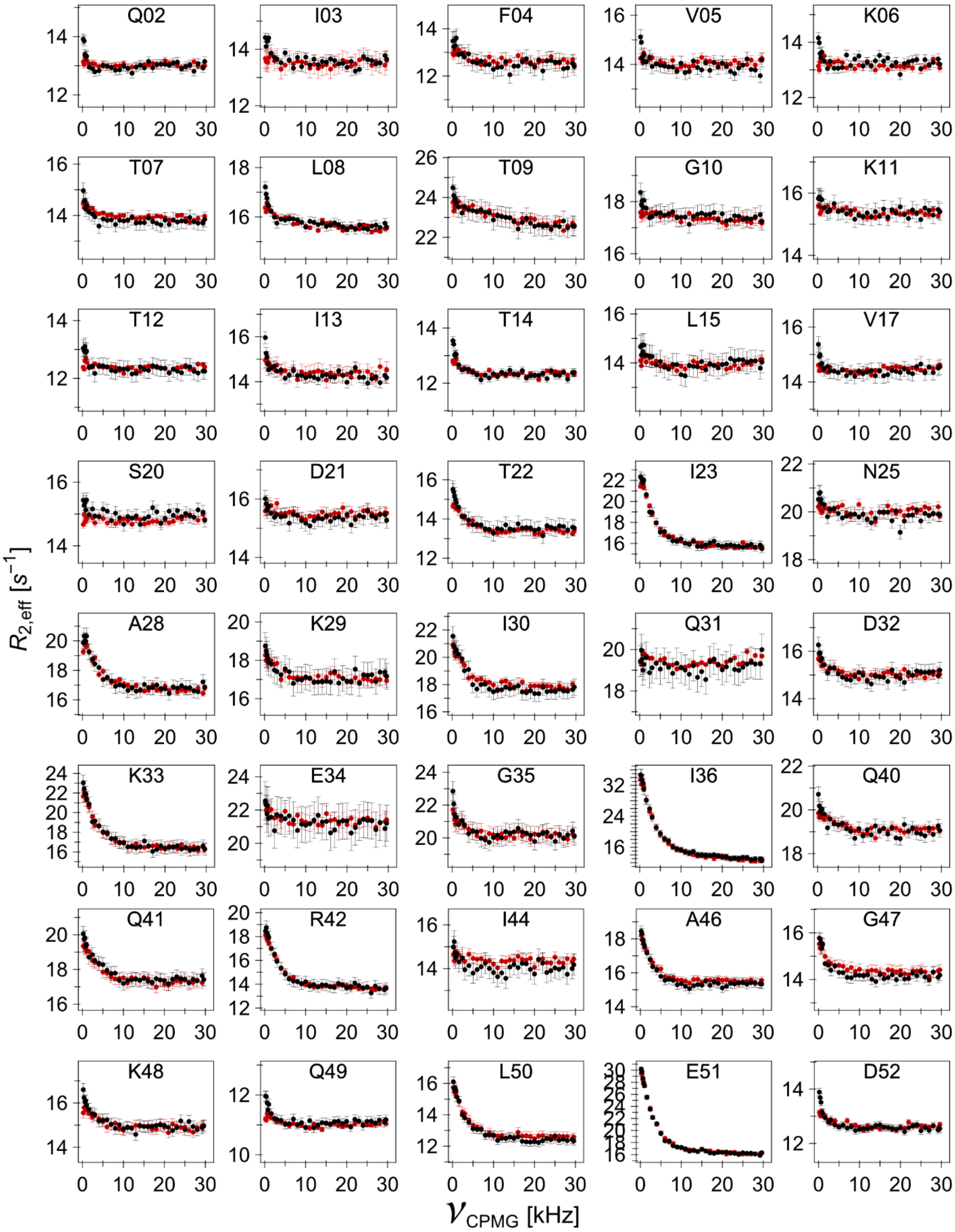

Figure contd.

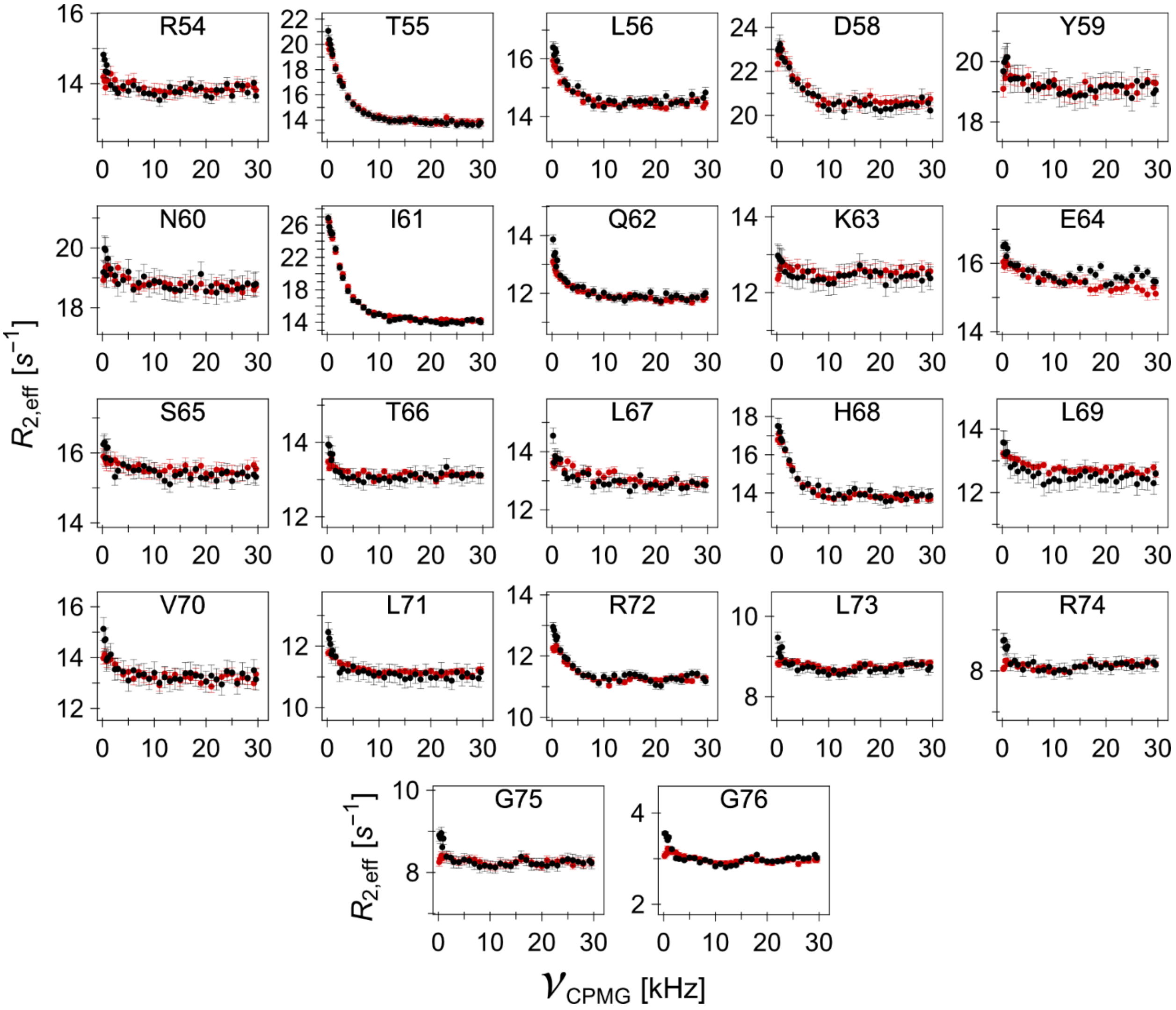

Fig. S2 Comparison of site-specific backbone ^1H_N E-CPMG relaxation dispersion curves of ubiquitin acquired using hard pulse (black) vs. amide selective REBURP refocusing pulse (red) in the rcINEPT element at 277 K under 600 MHz. Using the hard refocusing pulse was found to cause a minute pseudo dispersion of < 1 s^{-1}, spanning only the very low CPMG frequencies up to 500 Hz.

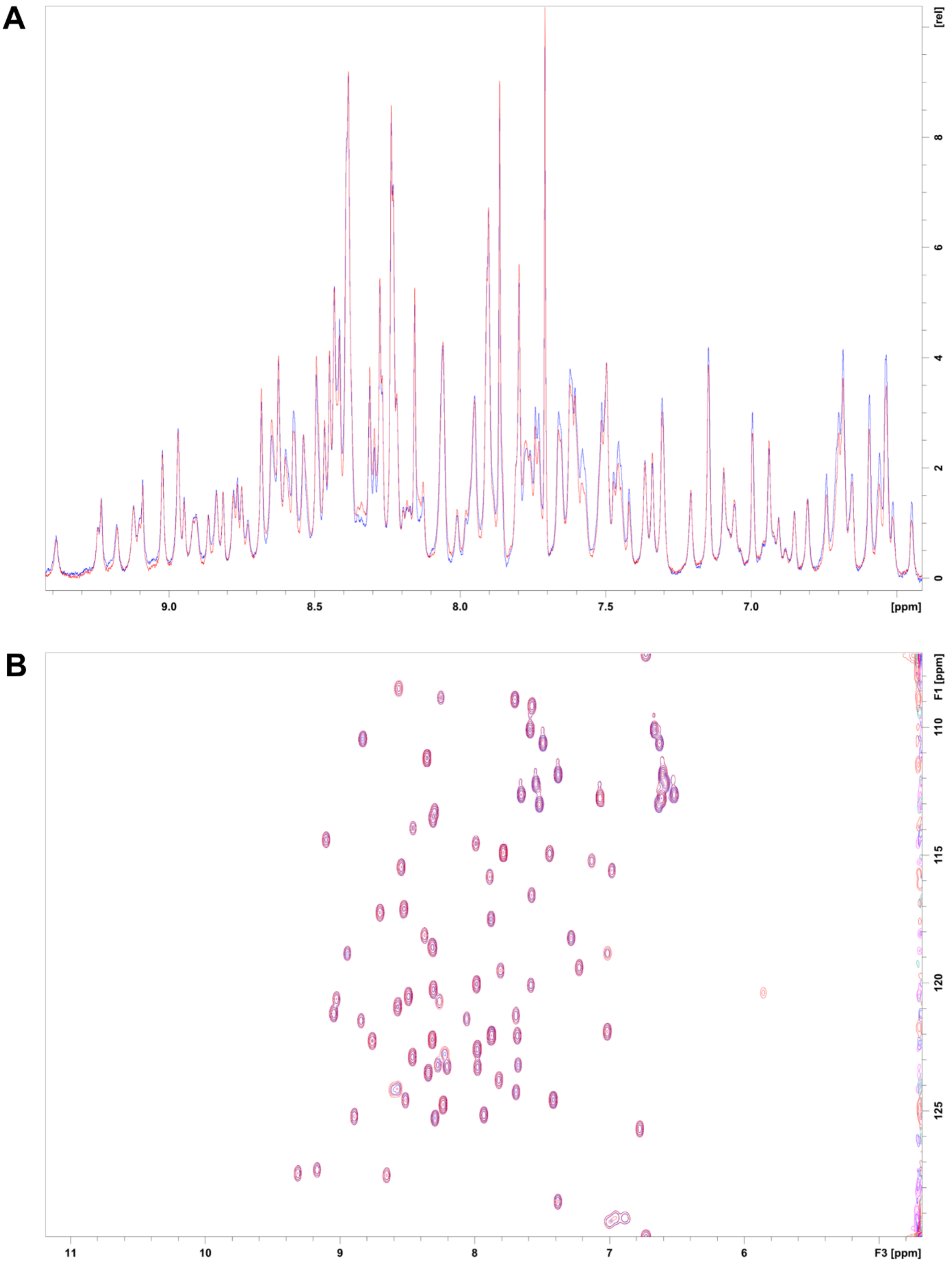
A
[rel]
8
6
4
2
0
9.0
8.5
8.0
7.5
7.0
[ppm]
B
F1 [ppm]
110
115
120
125
11
10
9
8
7
6
F3 [ppm]

C

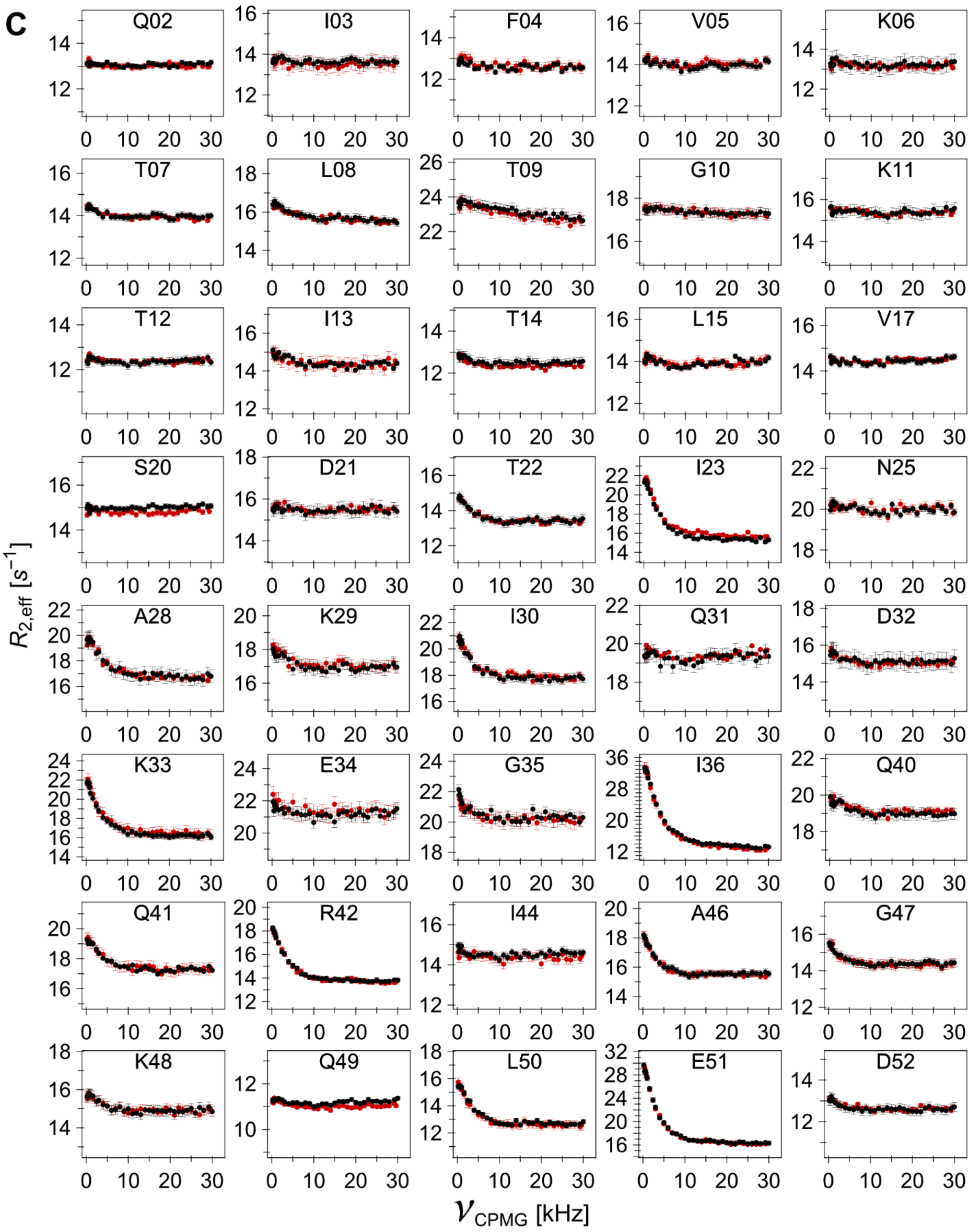

Figure contd.

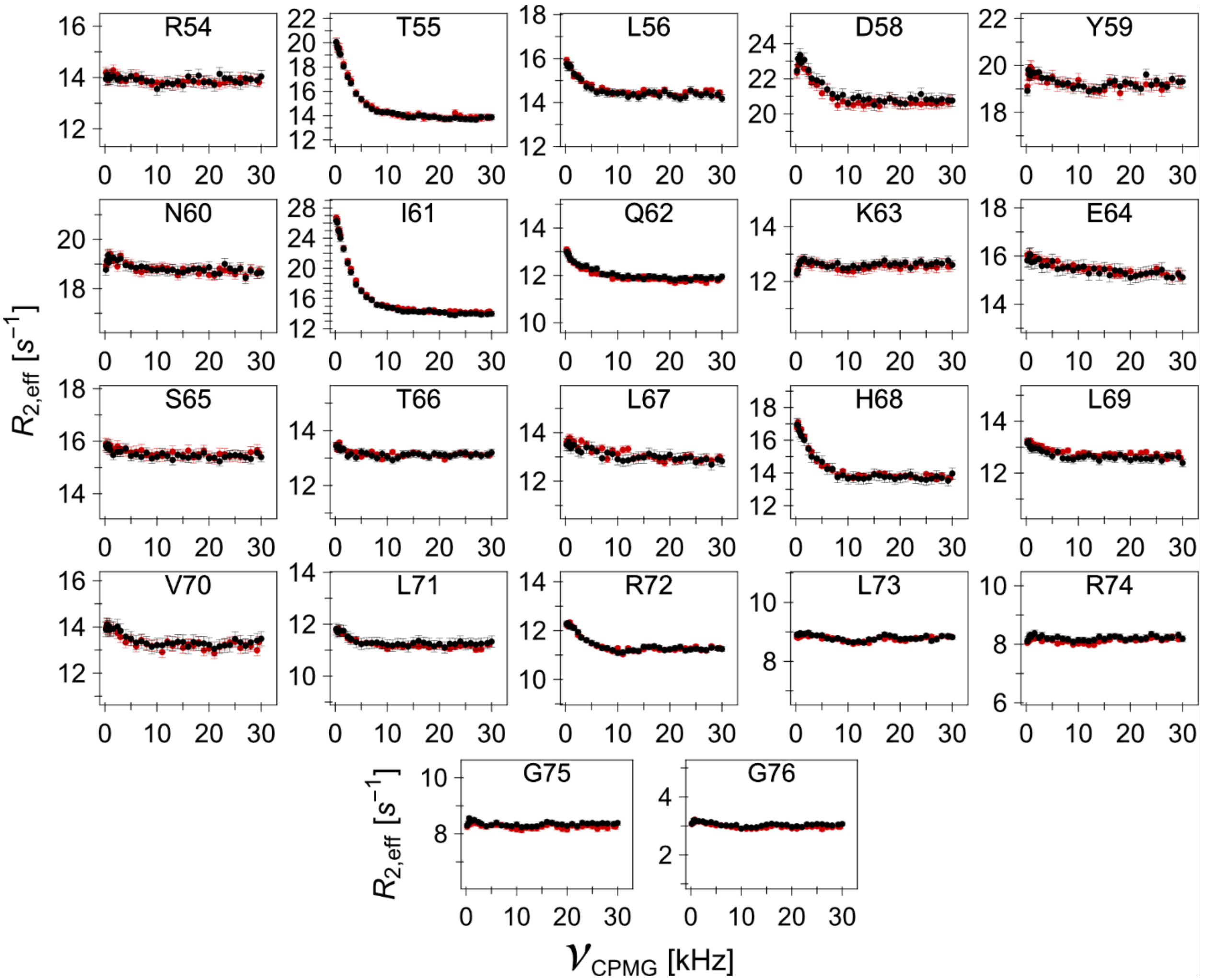

Fig. S3 (A) Overlay of 1D ^{1}H spectra acquired at 600 MHz, 277 K with the pulse sequence shown in Fig. 1 for CPMG frequencies of 200 Hz (blue) and 30 kHz (red), collected without heat compensation. The spectra were collected up to 1 s of acquisition with 16384 complex points during ^{1}H direct dimension to achieve high resolution. ^{15}N decoupling was turned off during acquisition to protect the probe. **(B)** Overlay of 2D ^{15}N-^{1}H slices from the dataset acquired at 800 MHz, 277 K with the pulse sequence shown in Fig. 1, corresponding to CPMG frequencies of 200 Hz (blue) and 32 kHz (red), collected with heat compensation. For overlay purposes the spectra have been minimally processed in topspin 4.0.8 **(C)** Comparison of site-specific backbone $^{1}H_{N}$ E-CPMG relaxation dispersion curves of ubiquitin acquired with (black) and without (red) activated

heat compensation element at 277 K, 600 MHz. The results were identical within experimental error, indicating minimal change on sample temperature from pulsing.

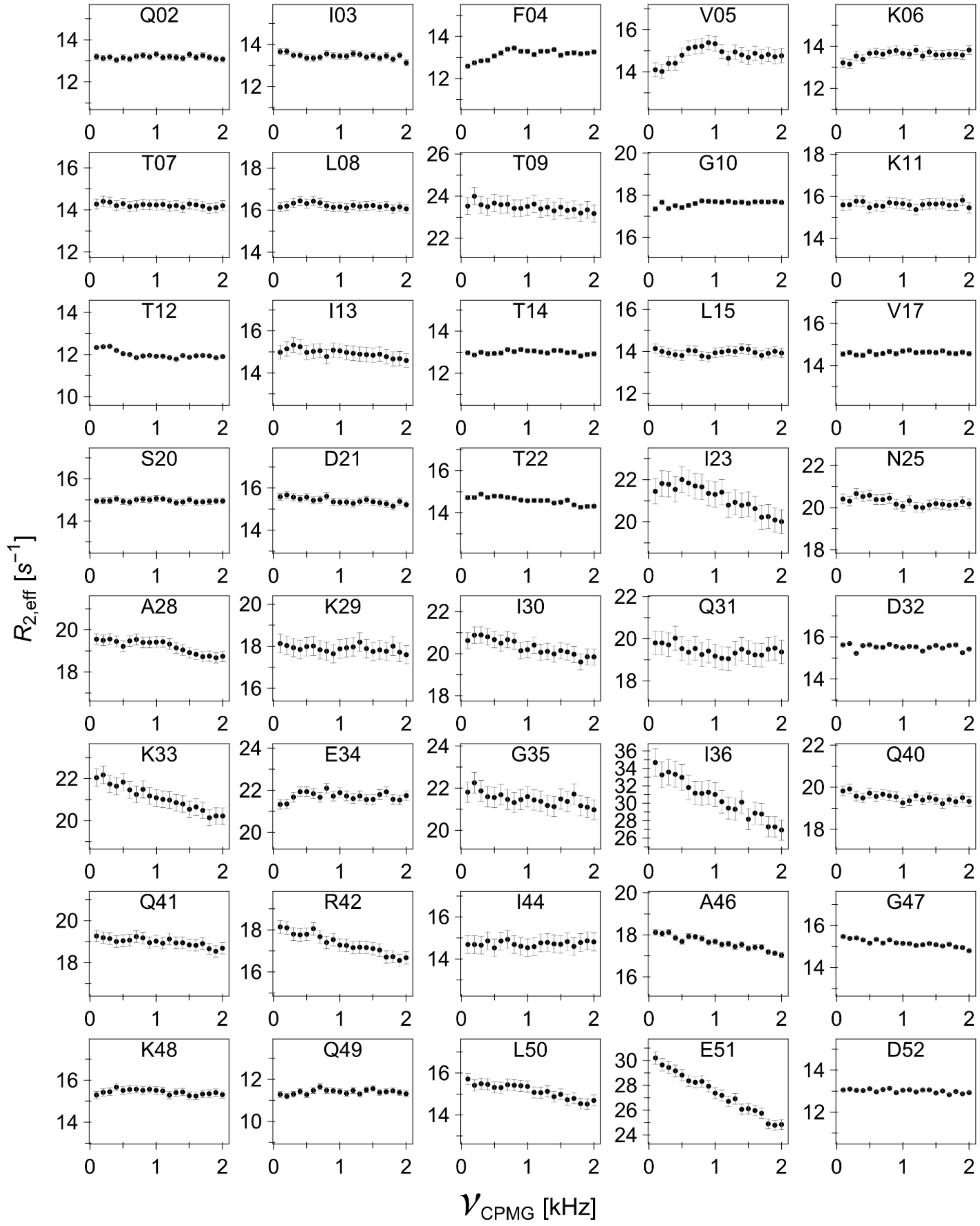

Figure contd.

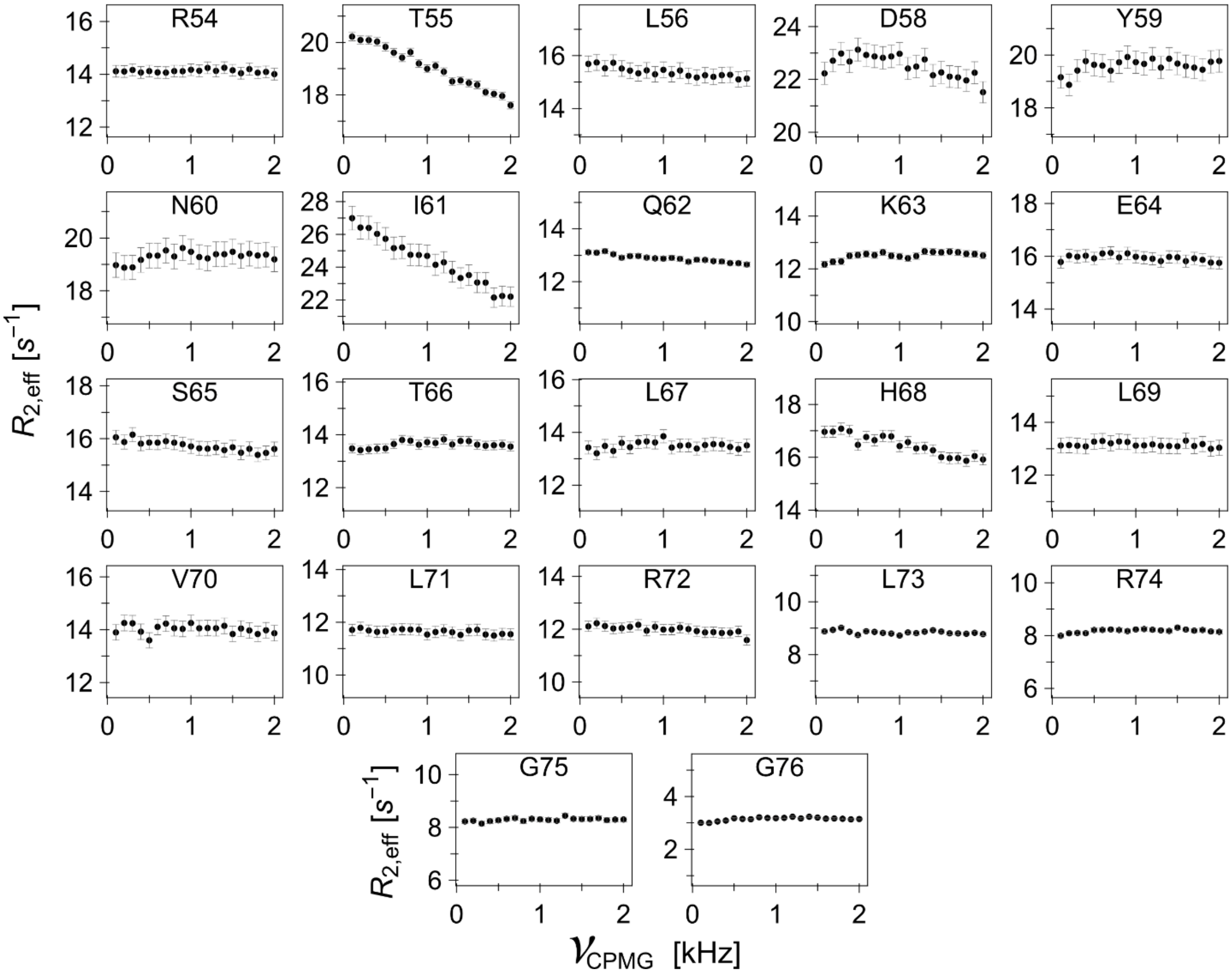

Fig. S4 Measurement of backbone $^{1}H_{N}$ site-specific relaxation dispersion curves of ubiquitin up to 2 kHz CPMG frequency obtained using the pulse sequence shown in Fig. 1 at 600 MHz, 277 K.

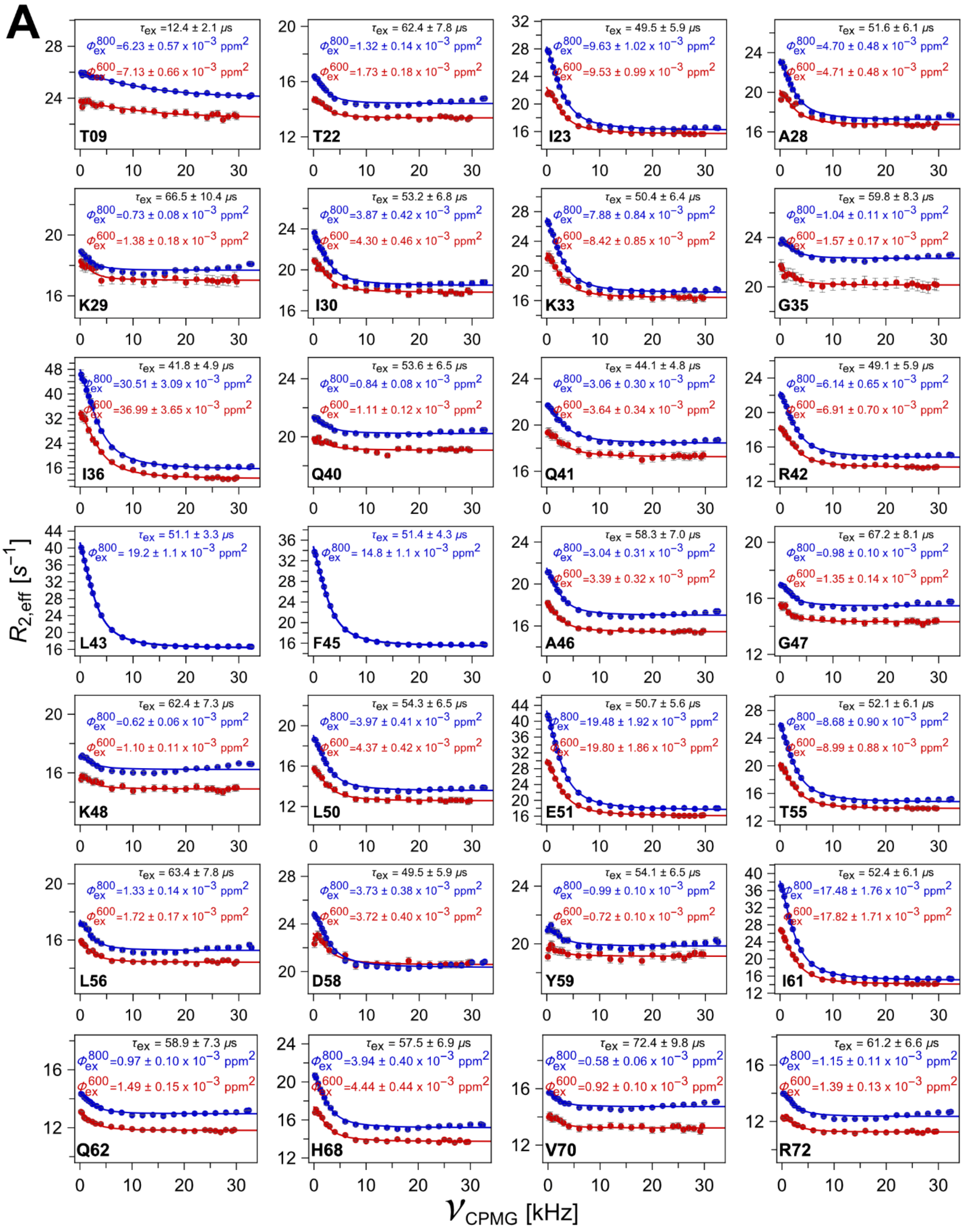
A
$R_{2,\mathrm{eff}}$ [s^{-1}]
ν_{CPMG} [kHz]
T09
τ_{ex} =12.4 ± 2.1 μs
Φ_{ex}^{800}=6.23 ± 0.57 x 10^{-3} ppm^2
Φ_{ex}^{600}=7.13 ± 0.66 x 10^{-3} ppm^2
T22
τ_{ex} = 62.4 ± 7.8 μs
Φ_{ex}^{800}=1.32 ± 0.14 x 10^{-3} ppm^2
Φ_{ex}^{600}=1.73 ± 0.18 x 10^{-3} ppm^2
I23
τ_{ex} = 49.5 ± 5.9 μs
Φ_{ex}^{800}=9.63 ± 1.02 x 10^{-3} ppm^2
Φ_{ex}^{600}=9.53 ± 0.99 x 10^{-3} ppm^2
A28
τ_{ex} = 51.6 ± 6.1 μs
Φ_{ex}^{800}=4.70 ± 0.48 x 10^{-3} ppm^2
Φ_{ex}^{600}=4.71 ± 0.48 x 10^{-3} ppm^2
K29
τ_{ex} = 66.5 ± 10.4 μs
Φ_{ex}^{800}=0.73 ± 0.08 x 10^{-3} ppm^2
Φ_{ex}^{600}=1.38 ± 0.18 x 10^{-3} ppm^2
I30
τ_{ex} = 53.2 ± 6.8 μs
Φ_{ex}^{800}=3.87 ± 0.42 x 10^{-3} ppm^2
Φ_{ex}^{600}=4.30 ± 0.46 x 10^{-3} ppm^2
K33
τ_{ex} = 50.4 ± 6.4 μs
Φ_{ex}^{800}=7.88 ± 0.84 x 10^{-3} ppm^2
Φ_{ex}^{600}=8.42 ± 0.85 x 10^{-3} ppm^2
G35
τ_{ex} = 59.8 ± 8.3 μs
Φ_{ex}^{800}=1.04 ± 0.11 x 10^{-3} ppm^2
Φ_{ex}^{600}=1.57 ± 0.17 x 10^{-3} ppm^2
I36
τ_{ex} = 41.8 ± 4.9 μs
Φ_{ex}^{800}=30.51 ± 3.09 x 10^{-3} ppm^2
Φ_{ex}^{600}=36.99 ± 3.65 x 10^{-3} ppm^2
Q40
τ_{ex} = 53.6 ± 6.5 μs
Φ_{ex}^{800}=0.84 ± 0.08 x 10^{-3} ppm^2
Φ_{ex}^{600}=1.11 ± 0.12 x 10^{-3} ppm^2
Q41
τ_{ex} = 44.1 ± 4.8 μs
Φ_{ex}^{800}=3.06 ± 0.30 x 10^{-3} ppm^2
Φ_{ex}^{600}=3.64 ± 0.34 x 10^{-3} ppm^2
R42
τ_{ex} = 49.1 ± 5.9 μs
Φ_{ex}^{800}=6.14 ± 0.65 x 10^{-3} ppm^2
Φ_{ex}^{600}=6.91 ± 0.70 x 10^{-3} ppm^2
L43
τ_{ex} = 51.1 ± 3.3 μs
Φ_{ex}^{800}= 19.2 ± 1.1 x 10^{-3} ppm^2
F45
τ_{ex} = 51.4 ± 4.3 μs
Φ_{ex}^{800}= 14.8 ± 1.1 x 10^{-3} ppm^2
A46
τ_{ex} = 58.3 ± 7.0 μs
Φ_{ex}^{800}=3.04 ± 0.31 x 10^{-3} ppm^2
Φ_{ex}^{600}=3.39 ± 0.32 x 10^{-3} ppm^2
G47
τ_{ex} = 67.2 ± 8.1 μs
Φ_{ex}^{800}=0.98 ± 0.10 x 10^{-3} ppm^2
Φ_{ex}^{600}=1.35 ± 0.14 x 10^{-3} ppm^2
K48
τ_{ex} = 62.4 ± 7.3 μs
Φ_{ex}^{800}=0.62 ± 0.06 x 10^{-3} ppm^2
Φ_{ex}^{600}=1.10 ± 0.11 x 10^{-3} ppm^2
L50
τ_{ex} = 54.3 ± 6.5 μs
Φ_{ex}^{800}=3.97 ± 0.41 x 10^{-3} ppm^2
Φ_{ex}^{600}=4.37 ± 0.42 x 10^{-3} ppm^2
E51
τ_{ex} = 50.7 ± 5.6 μs
Φ_{ex}^{800}=19.48 ± 1.92 x 10^{-3} ppm^2
Φ_{ex}^{600}=19.80 ± 1.86 x 10^{-3} ppm^2
T55
τ_{ex} = 52.1 ± 6.1 μs
Φ_{ex}^{800}=8.68 ± 0.90 x 10^{-3} ppm^2
Φ_{ex}^{600}=8.99 ± 0.88 x 10^{-3} ppm^2
L56
τ_{ex} = 63.4 ± 7.8 μs
Φ_{ex}^{800}=1.33 ± 0.14 x 10^{-3} ppm^2
Φ_{ex}^{600}=1.72 ± 0.17 x 10^{-3} ppm^2
D58
τ_{ex} = 49.5 ± 5.9 μs
Φ_{ex}^{800}=3.73 ± 0.38 x 10^{-3} ppm^2
Φ_{ex}^{600}=3.72 ± 0.40 x 10^{-3} ppm^2
Y59
τ_{ex} = 54.1 ± 6.5 μs
Φ_{ex}^{800}=0.99 ± 0.10 x 10^{-3} ppm^2
Φ_{ex}^{600}=0.72 ± 0.10 x 10^{-3} ppm^2
I61
τ_{ex} = 52.4 ± 6.1 μs
Φ_{ex}^{800}=17.48 ± 1.76 x 10^{-3} ppm^2
Φ_{ex}^{600}=17.82 ± 1.71 x 10^{-3} ppm^2
Q62
τ_{ex} = 58.9 ± 7.3 μs
Φ_{ex}^{800}=0.97 ± 0.10 x 10^{-3} ppm^2
Φ_{ex}^{600}=1.49 ± 0.15 x 10^{-3} ppm^2
H68
τ_{ex} = 57.5 ± 6.9 μs
Φ_{ex}^{800}=3.94 ± 0.40 x 10^{-3} ppm^2
Φ_{ex}^{600}=4.44 ± 0.44 x 10^{-3} ppm^2
V70
τ_{ex} = 72.4 ± 9.8 μs
Φ_{ex}^{800}=0.58 ± 0.06 x 10^{-3} ppm^2
Φ_{ex}^{600}=0.92 ± 0.10 x 10^{-3} ppm^2
R72
τ_{ex} = 61.2 ± 6.6 μs
Φ_{ex}^{800}=1.15 ± 0.11 x 10^{-3} ppm^2
Φ_{ex}^{600}=1.39 ± 0.13 x 10^{-3} ppm^2

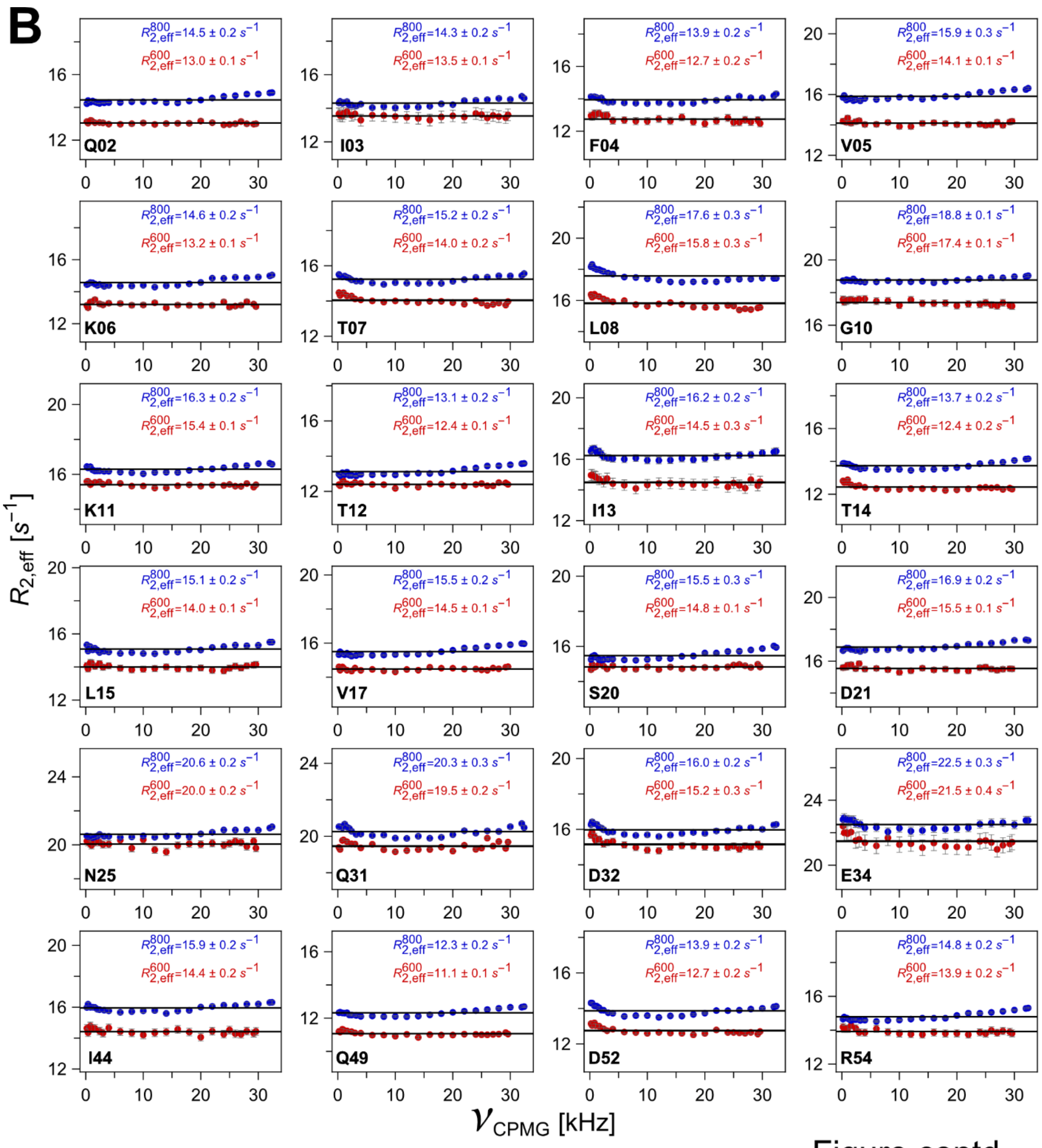

Figure contd.

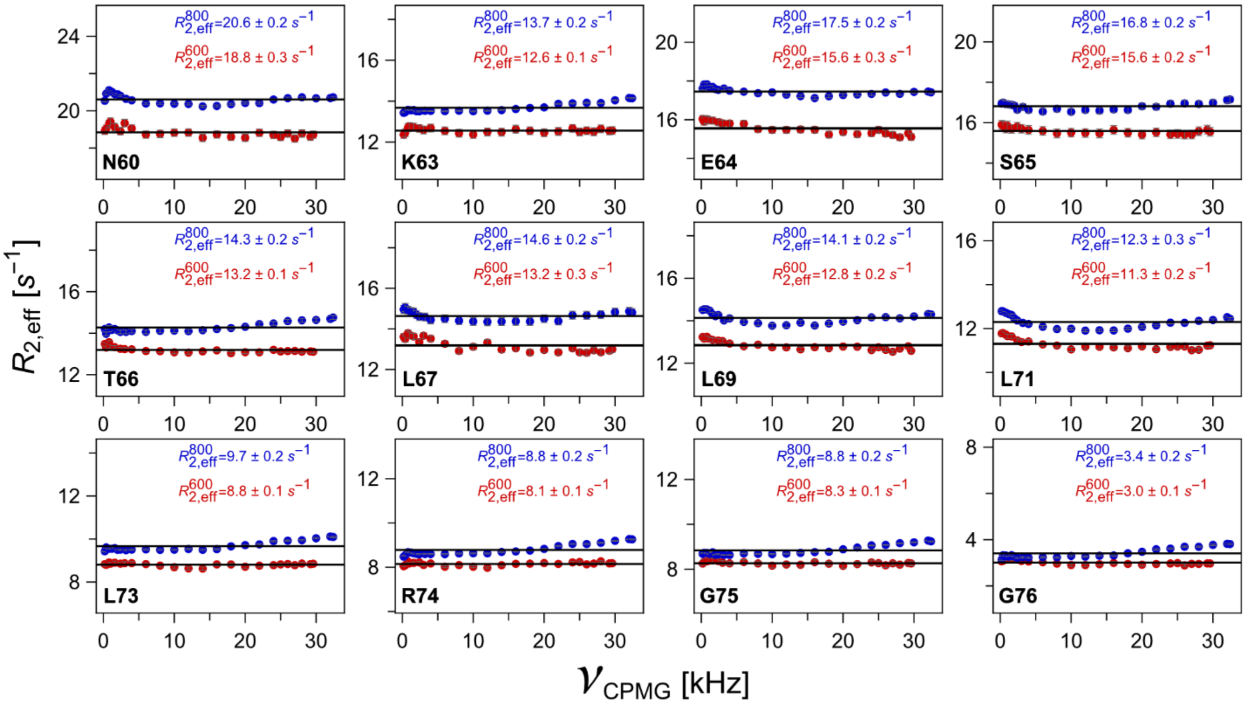

Fig. S5 Measurement of site-specific backbone $^{1}H_{N}$ E-CPMG relaxation dispersion curves of ubiquitin measured up to 30 kHz CPMG frequency at 277 K, 600 MHz (red), and 800 MHz (blue) from all resolved sites. (A) Dispersion profiles from all residues with the presence of fast (μs) dynamics with the fitted site-specific motional timescales indicated inside the figures (black). The fitted field-specific chemical shift variance values at 600 MHz (red) and 800 MHz (blue) are also shown. Residues L43 and F45 could only be resolved in the 800 MHz data set at 277 K. (B) Dispersion profiles from sites without detectable fast (μs) dynamics. Black lines indicate the average $R_{2,\mathrm{eff}}$ values computed over the whole CPMG frequency range. The same average $R_{2,\mathrm{eff}}$ values along with computed RMSDs are represented in the figure color-coded according to magnetic field strength. Residues T07, L08, I13, D32, E34, D52, L67, L69, and L71 show the possible presence of a fast (μs) exchange process, although the data has not been fit.

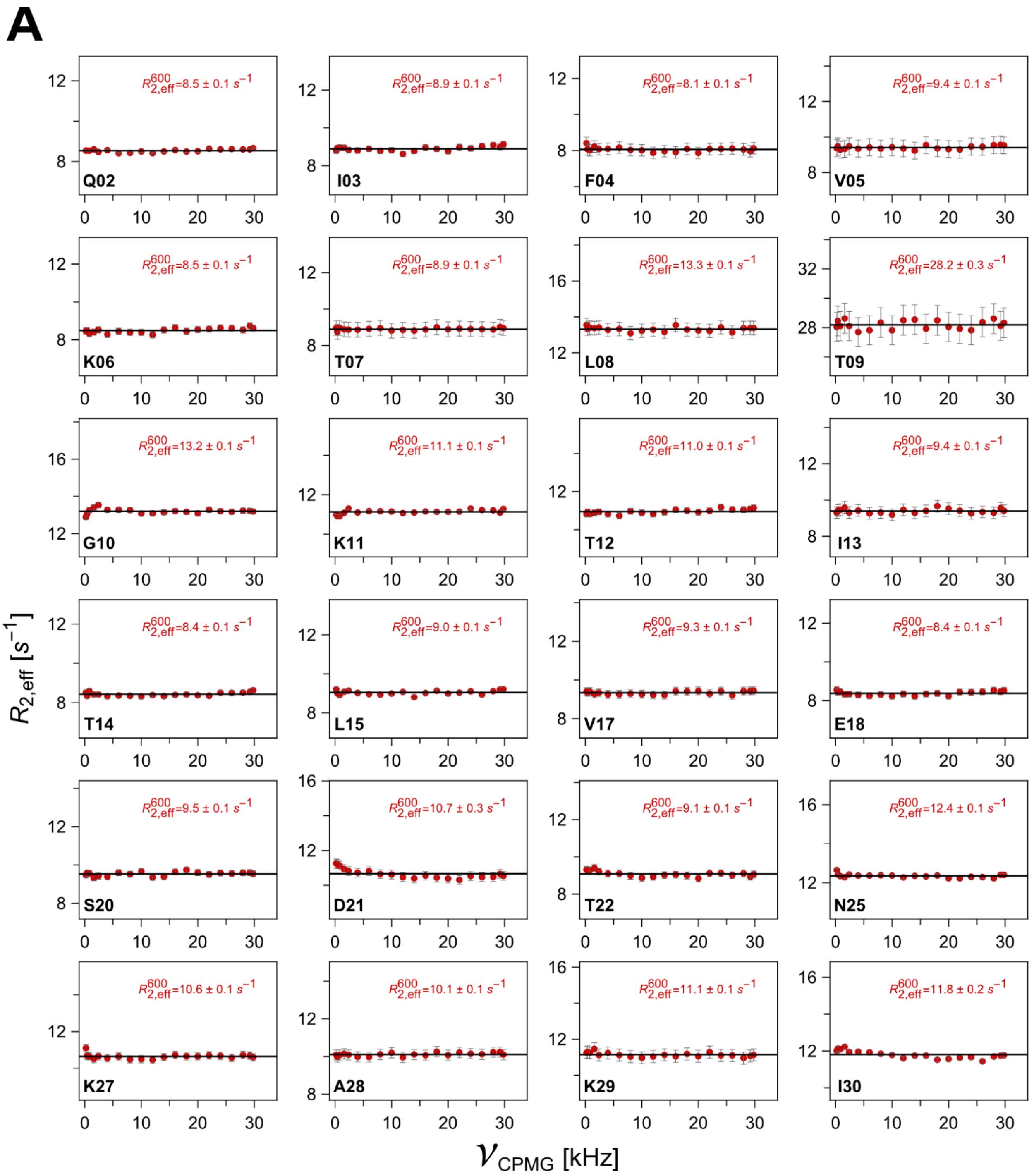

Figure contd.

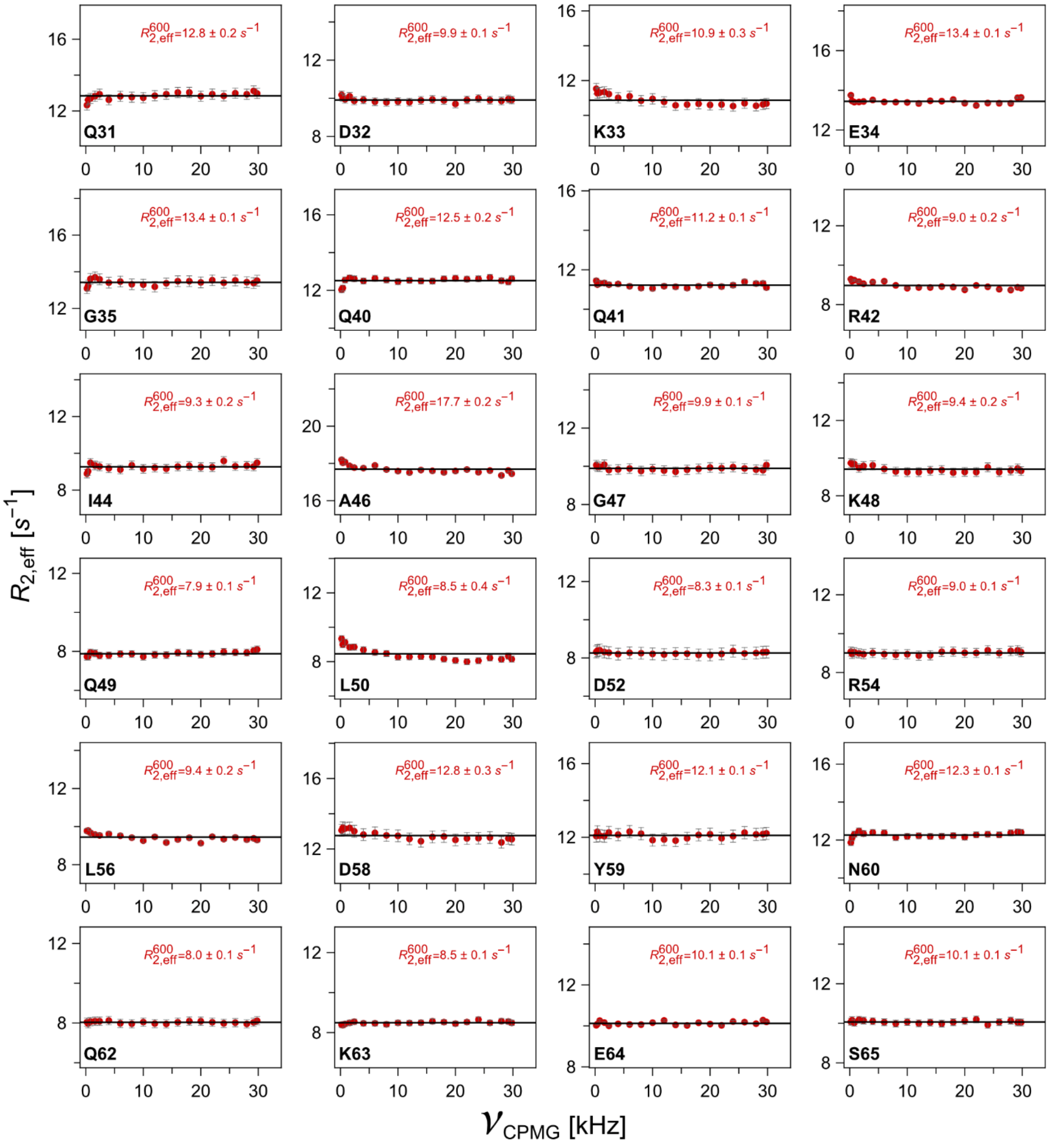

Figure contd.

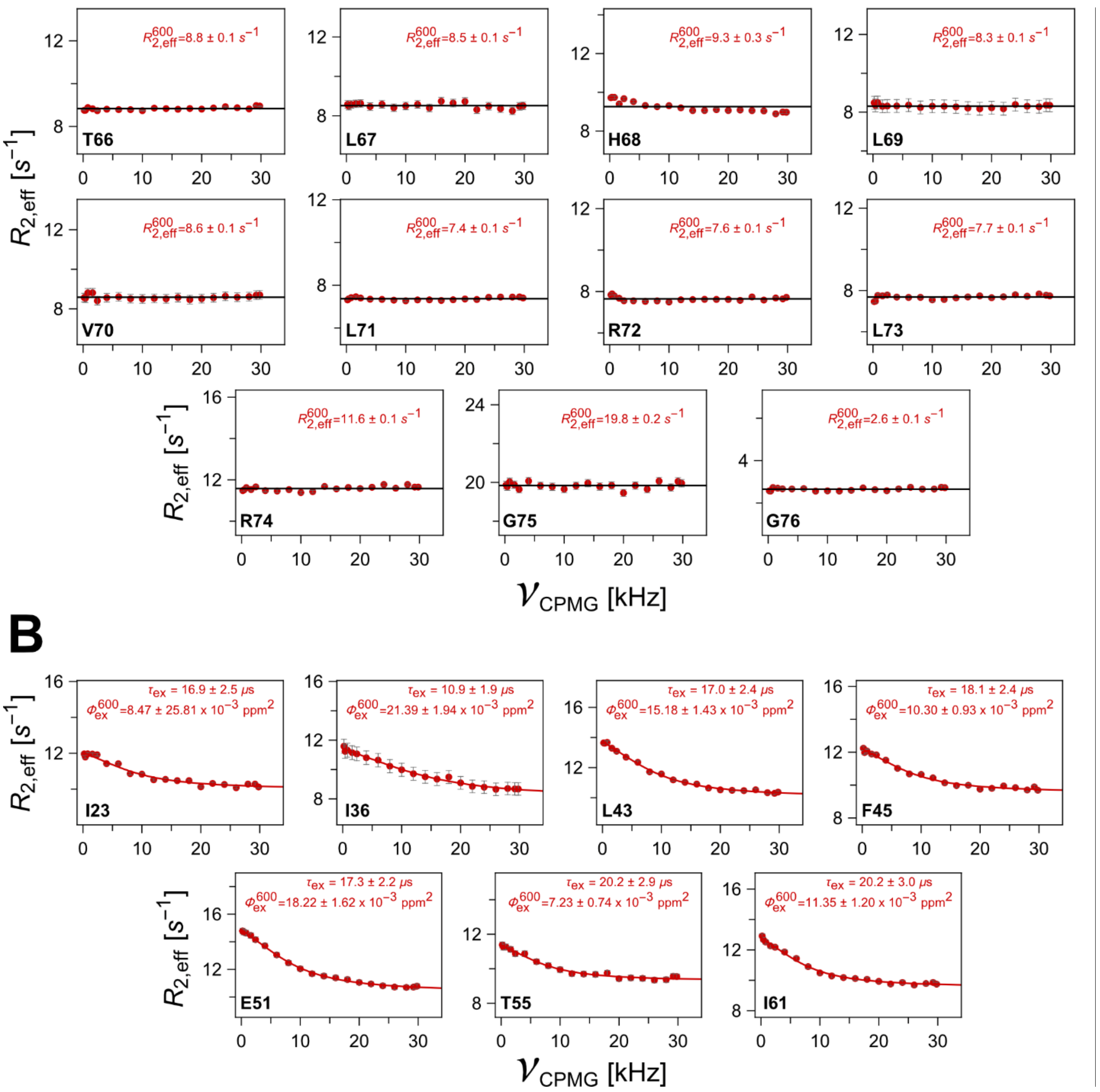

Fig. S6 Measurement of site-specific backbone ^1H_N E-CPMG relaxation dispersion curves of ubiquitin measured up to 30 kHz CPMG frequency at 292 K, 600 MHz (red) from all resolved sites. (A) Dispersion profiles from sites without detectable fast (μs) dynamics. Black lines indicate the average $R_{2,\mathrm{eff}}$ values computed over the whole CPMG frequency range. The same average $R_{2,\mathrm{eff}}$ values along with computed RMSDs are represented in the figure. Residues K33, L50, and H68 show detectable dispersion, although the data has not been fit. (B) Dispersion profiles obtained at

600 MHz, 292 K from all residues with the presence of fast (μs) dynamics with the fitted site-specific motional timescales and chemical shift variance values indicated in the figures (red).

References

Ishima R, Torchia DA (2003) Extending the range of amide proton relaxation dispersion experiments in proteins using a constant-time relaxation-compensated CPMG approach. J Biomol NMR 25:243–248.

Pratihar S (2022). Detecting the undetectable: Functional protein motions in the hidden timescale window revealed by NMR relaxation measurements, Georg-August-University Göttingen.

Yip GNB, Zuiderweg ERP (2004) A phase cycle scheme that significantly suppresses offset-dependent artifacts in the R_2-CPMG ^{15}N relaxation experiment. J Magn Reson 171:25–36.

Yuwen T, Kay LE (2019) Revisiting $^{1}H^{N}$ CPMG relaxation dispersion experiments: a simple modification can eliminate large artifacts. J Biomol NMR 73:641–650.

```
/* 1H E-CPMG Version for Bruker Avance Neo Console (Also runs on  Avance III HD)
        Pulse seq elements/syntax based on ref:
        Yuwen and Kay, JBNMR, 2019 (used as a starting point)
        Ishima and Torchia, JBNMR, 2003
    - This seq. goes up to the 1H hard pulse limit. USE WITH CAUTION.
    - Many safety switches modified.
    - Relaxation compensation element with default Reburp refocusing.

    FnMODE: F1-States, F2-QF

    Run Options: Dreburp: REBURP rcINEPT
                 DH_heat: turn on heat comp
                 Df1180: -90/180 phase correction

    max number of loop counter list = l8 < l13 (MUST SET THIS PROPERLY)

    list elements divisible by 16 or 8 or 4 (in decreasing order of preference)

*/

prosol relations=<triple>

#include <Avance.incl>
#include <Grad.incl>
#include <Delay.incl>

/* Reburp parameter calc */

  "p16=4.875/(cnst2*bf1/1e6)"   /* REBURP pulse length  */
  "spw16=plw1*(pow((p1*2.0/p16)/0.07981,2))"   /* REBURP power level  */
  "spoff16=0"
  "spoal16=0.5"

/*    Delays       */

define delay taua
  "taua=d3"              /* 2.25m use JNH=105 to decreas the tauxh duration */
                         /* 1 / 4J(XH)              */

define delay taub
  "taub=d4"              /* 2.68m use JNH=105 to decreas the tauxh duration */
                         /* 1 / 4J(XH)              */

define delay time_T2
  "time_T2=d17"

define delay tauCPMG

define delay tau_wgate
    "tau_wgate = abs(1.0/(4.0*cnst1))*2.0"

"d11=30m"
"in0=inf1/2"             /* t1/2 increment */
"TAU2=0.2u"


/*    Pulses      */

define pulse pwh
  "pwh=p1"               /* 1H hard pulse at power level pl1 */

define pulse pwn
  "pwn=p3"               /* PW90 for N pulse at power level pl3 */

define pulse pwh_cp
  "pwh_cp=p1"          /* 1H 90 degree pulse for 1H CPMG at pl1, set to p1 */

define pulse pwh_reb
  "pwh_reb=p16"        /* 1H reburp 180 pulse at pl16 */
```

```
#ifdef H_heat
  define pulse pw_sl
    "pw_sl=p12"
#endif

/* f1180: Start t1 at half dwell to get -90/180 phase correction */

#ifdef f1180
  "d0=(in0/2)"
#else
  "d0=(0.2u/2)"
#endif


/*    Loop counters     */

define list<loopcounter> ncyc_cp=<$VCLIST>

define loopcounter ncyc_cp_max
  "ncyc_cp_max=l8"            /* max value of ncyc used */



/* Assign cnsts to check validity of parameter range */

"cnst15=plw1"          /* set max at 18 W */
"cnst3=plw3"           /* set max at 150 W */
"cnst31=plw31"         /* set max at 15W */


/* Define CPMG pulses */

#define cpmg_F if "nsdone%2 == 0" {\n (pwh_cp*2.0 ph11):f1 \n}\n else {\n (pwh_cp*2.0 ph12):f1 \n}
#define cpmg_R if "nsdone%2 == 0" {\n (pwh_cp*2.0 ph21):f1 \n}\n else {\n (pwh_cp*2.0 ph22):f1 \n}


/* Initialize variables */

"l2=0"
"l4=0"

"l13 = time_T2*0.25/pwh"
"l14 = 0"

"acqt0=0"
baseopt_echo


/* BEGIN ACTUAL PULSE SEQUENCE */

1 ze

/* Check validity of parameters and assign values to some of them */

  if "cnst15 > 18" {
    2u
    print "error: pl1 too large !!!  "
    goto HaltAcqu
  }

  if "cnst3 > 150" {
    2u
    print "error: pl3 too large !!!  "
    goto HaltAcqu
  }

  if "cnst31 > 15" {
    2u
    print "error: pl31 too large !!!  "
    goto HaltAcqu
  }
```

```
  if "time_T2 > 40m" {
    2u
    print "error:time_T2 too long"
    goto HaltAcqu
  }

  if "aq > 50m" {
    2u
    print "error: aq is too long"
    goto HaltAcqu
  }

2 d11 do:f3

/* Update list pointers */

  2u
  "ncyc_cp.idx=l2"
  2u rpp11 rpp12 rpp21 rpp22

/* Continue to check real time variables  */

 "l4 = (trunc(ncyc_cp + 0.3))"

 "l14 = (trunc(ncyc_cp_max + 0.3) - l4)"        /* heat comp */

  2u

  if "ncyc_cp > 0" {
    "tauCPMG = time_T2*0.25/ncyc_cp - pwh_cp*0.75"

    if "tauCPMG + pwh_cp < pwh" {
      2u
      print "error: tauCPMG < 0"
      goto HaltAcqu
    }
  }

  if "ncyc_cp_max > l13" {
    2u
    print "error: tauCPMG < 0"
    goto HaltAcqu
  }

  if "ncyc_cp > ncyc_cp_max" {
    2u
    print "error: ncyc_cp_max must be greater than or equal to ncyc_cp !!!"
    goto HaltAcqu
  }

/* 1H Heating period */

  4u pl1:f1

#ifdef H_heat
  4u
  (pw_sl:sp1 ph27):f1
  4u pl1:f1


if "l14 > 0" {
7 (pwh_cp*2.0 ph26):f1
  (pwh_cp*2.0 ph28):f1
  lo to 7 times l14
  }

  4u
  (pw_sl:sp1 ph29):f1
  4u pl1:f1
```

```
#endif

/* Destroy residual 1H magnetization prior to d1 */

  4u pl1:f1              /* power pl1 for 1H pulses */
  20u UNBLKGRAD

  (pwh ph26):f1

  2u
  p51:gp1*0.5
  d16

  (pwh ph27):f1

  2u
  p51:gp1
  d16

  4u BLKGRAD

  d1

/* Eliminate equilibrium magnetization on 15N */

  20u UNBLKGRAD

  4u pl3:f3
  (pwn ph26):f3

  2u
  p51:gp1
  d16

/* This is the real start */

  (pwh ph26):f1

  2u
  p52:gp2
  d16

  "DELTA = taua - 2u - p52 - d16"
  DELTA

  (center (pwh*2.0 ph26):f1 (pwn*2.0 ph26):f3)

  DELTA

  2u
  p52:gp2
  d16

  (pwh ph27):f1

  2u
  p53:gp3
  d16

  (pwn ph1):f3

  "TAU1=larger(d0-pwn*2.0/PI-pwh*2.0, TAU2)"

  TAU1
  (pwh ph27 pwh*2.0 ph26 pwh ph27):f1
  TAU1

  (pwn ph26):f3

  2u
  p54:gp4
```

```
  d16

  10u fq=cnst1(sfo hz):f1

/* First half of CPMG relaxation period */

  (pwh_cp ph26):f1

  if "l4 > 0" {
5     tauCPMG
      cpmg_F
      tauCPMG ipp11 ipp12 ipp21 ipp22
      lo to 5 times l4
  }

/*  REBURP RC-INEPT */

#ifdef reburp
  2u
  p52:gp7
  d16

  "DELTA = taub - 2u - p52 - d16 - pwh_reb*0.5 - 4u"
  DELTA

  4u
  (center (pwh_reb:sp16 ph2:r):f1 (pwn*2.0 ph26):f3)
  4u pl1:f1

  DELTA

  2u
  p52:gp7
  d16
#else
  2u
  p52:gp7
  d16

  "DELTA = taub - 2u - p52 - d16 - pwn"
  DELTA

  (center (pwh*2.0 ph2:r):f1 (pwn*2.0 ph26):f3)

  DELTA

  2u
  p52:gp7
  d16
#endif

/* Second half of CPMG relaxation period */

  if "l4 > 0" {
6     tauCPMG dpp11 dpp12 dpp21 dpp22
      cpmg_R
      tauCPMG
      lo to 6 times l4
  }

  (pwh_cp ph27):f1

/* Compensation for R1 relaxation during CPMG pulses */

  "DELTA = (ncyc_cp_max - ncyc_cp) * pwh_cp + 2u"
  DELTA

  2u
  p55:gp5
  d16
```

```
  10u fq=0(sfo hz):f1


  (pwh ph26):f1

  2u
  p56:gp6
  d16

  "DELTA = 4u + 4u + de"
  DELTA

  (pwh*0.231 ph27):f1
  tau_wgate
  (pwh*0.692 ph27):f1
  tau_wgate
  (pwh*1.462 ph27):f1
  tau_wgate
  (pwh*1.462 ph29):f1
  tau_wgate
  (pwh*0.692 ph29):f1
  tau_wgate
  (pwh*0.231 ph29):f1

  "DELTA = pwh*2.0/PI"
  DELTA

  2u
  p56:gp6
  d16

  4u pl31:f3
  4u BLKGRAD

/*   Signal detection and looping      */

  go=2 ph31 cpds3:f3
  d11 do:f3 mc #0 to 2
    F2QF(calclc(l2, 1))
    F1PH(calph(ph1, +90), caldel(d0, +in0) & calph(ph1, +180) & calph(ph31, +180))
HaltAcqu, 1m
exit

ph1=0 2 2 0 2 0 0 2
ph2 = 0 0 0 0 2 2 2 2 2 2 2 2 0 0 0 0

ph11=1 1 2 0 1 1 0 2 1 1 0 2 1 1 2 0
ph12=2 0 3 3 0 2 3 3 0 2 3 3 2 0 3 3
ph21=2 2 1 3 2 2 3 1 2 2 3 1 2 2 1 3
ph22=1 3 0 0 3 1 0 0 3 1 0 0 1 3 0 0
ph26=0
ph27=1
ph28=2
ph29=3
ph31=0 2 2 0 2 0 0 2

;d1: Repetition delay d1
;d3: taua (~ 2.4ms < 1/4JNH)
;d4: taub (~ 2.68 ms ~ 1/4JNH)
;d11: delay for disk i/o, 30ms
;d16: gradient recovery delay, 200us
;d17: Tcpmg
;pl1: power level for H90 pwh
;pl3: power level for N90 pwn
;pl31:power level for 15N decoupling
;sp1: power level for water Flipback 90 pulse
;spnam1: shape for water Flipback 90 pulse
;spnam16: Reburp.1000
;p1: 1H 90 degree pulse
;p3: 15N 90 degree pulse
;p12: water Flipback 90 pulse length
```

```
;p16: Reburp 180 1H
;p51: gradient pulse 51                                [1000 usec]
;p52: gradient pulse 52                                [500 usec]
;p53: gradient pulse 53                                [1000 usec]
;p54: gradient pulse 54                                [800 usec]
;p55: gradient pulse 55                                [1000 usec]
;p56: gradient pulse 56                                [800 usec]
;pcpd3 : 15N 90 degree pulse at power pl31 for 15N decouling during acqt
;cnst1: set to center of NHs - o1 (Hz)
;cnst2: bandwidth (ppm) of NHs region
;l4: ncyc_cp (filled from list)
;l8: ncyc_cp_max from vclist (MUST BE SET PROPERLY!)
;l13: max possible val of l4 given Tcpmg, l8 < l13 !!!
;l14: heat comp counter, automatically calculated
;vclist: variable counter list for ncyc_cp
;inf1: 1/SW(X) = 2*DW(X)
;in0: 1/(2*SW(x))=DW(X)
;nd0: 2
;ns: 4*n
;FnMODE: States in F1
;FnMODE: QF in F2

;gpz1: 15%
;gpz2: 20%
;gpz3: 30%
;gpz4: 50%
;gpz5: 40%
;gpz6: 80%
;gpz7: 24% (RC-INEPT)
;gpnam1: SMSQ10.32
;gpnam2: SMSQ10.32
;gpnam3: SMSQ10.32
;gpnam4: SMSQ10.32
;gpnam5: SMSQ10.32
;gpnam6: SMSQ10.32
;gpnam7: SMSQ10.32
;zgoptns: DH_heat, Dreburp, Df1180
```
